# Multispecific DNA coatings for self-assembly

**DOI:** 10.1039/d6sm00340k

**Published:** 2026-06-30

**Authors:** Tine C. M. Stevens, Amy van der Sluis, Ilja K. Voets, Pepijn G. Moerman

**Affiliations:** a Laboratory of Self-Organizing Soft Matter, Department of Chemical Engineering and Chemistry & Institute for Complex Molecular Systems, Eindhoven University of Technology P.O. Box 513 5600 MB Eindhoven The Netherlands p.g.moerman@tue.nl

## Abstract

DNA-coated particles are promising building blocks for functional and finite-sized assemblies because they can be programmed to exhibit orthogonal interactions through the sequence-specific hybridization of DNA strands. To fully exploit this programmability, it is important to develop particles with coatings that incorporate multiple distinct DNA sequences in tunable ratios and to understand how the coating composition influences self-assembly. Here, we compare two strategies for grafting multiple DNA sequences in tunable and well-defined ratios onto micron-sized colloidal particles. We found that a method based on click chemistry yielded mixed coatings with large batch-to-batch variation in composition, while a method based on isothermal DNA polymerization produced coatings with predictable compositions and a precision of a few percent, but it requires reaction rate measurements for each new sequence in the coating. Our self-assembly experiments showed that, even with precise control over coating composition, equilibrium co-assembly of multiple types of DNA-coated particles is limited by the number of interactions that are reversible within the same narrow temperature window. This finding highlights the need to explicitly incorporate sequential assembly pathways into structure design, with coating composition dictating the order of binding events. Together, our results show how systematic tuning of interaction strength and sequential assembly through multispecific DNA coatings is a prerequisite for the experimental realization of finite-sized and dynamic structures that have so far remained largely theoretical.

## Abstract 2

We present a strategy for coating colloids with multiple DNA sequences at defined ratios, enabling programmable binding to multiple partners.

## Graphical abstract

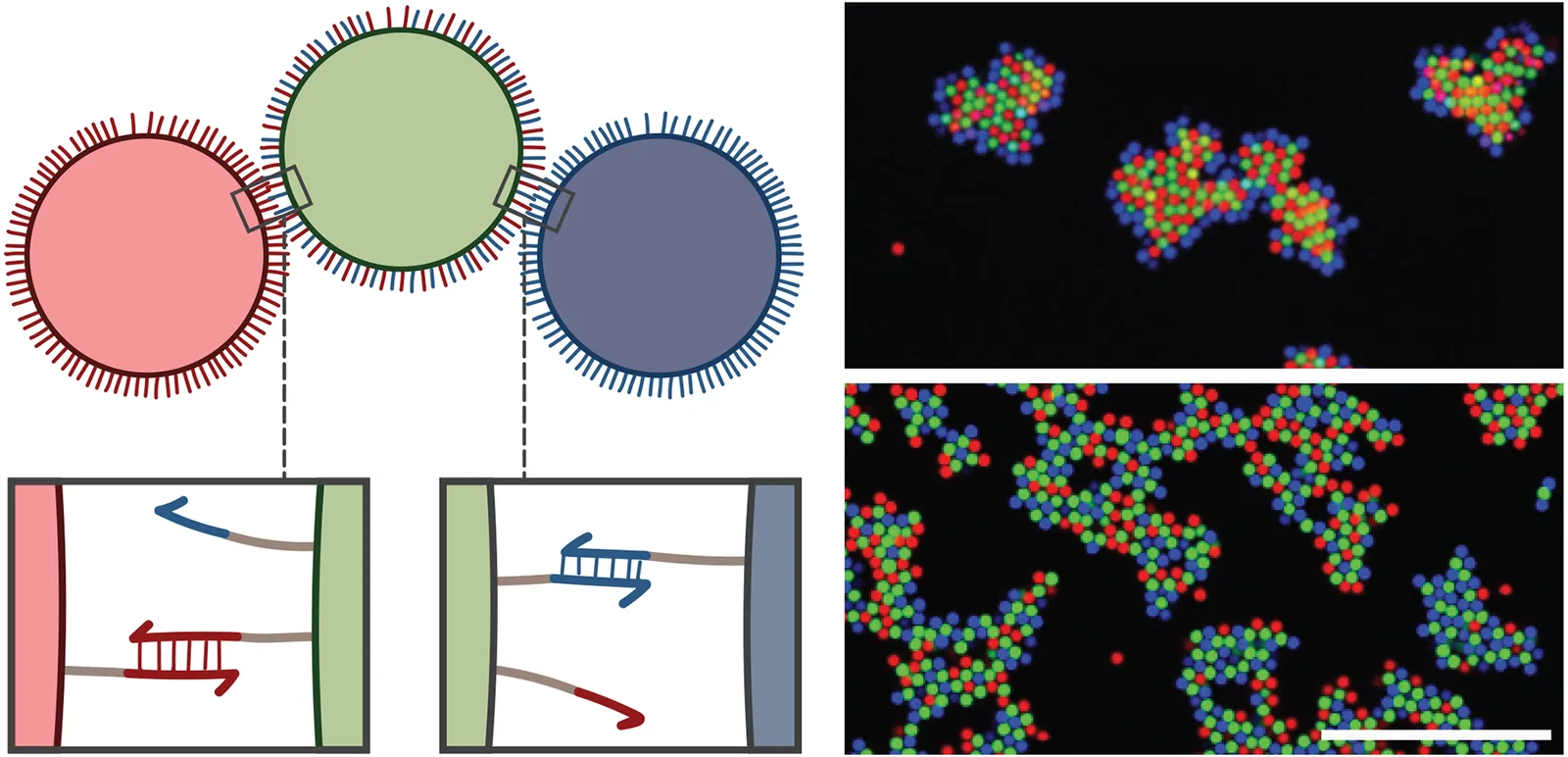

## 1 Introduction

DNA-coated colloids are versatile building blocks for programmable self-assembly on the nano- and micrometer scale with the ability to form complex, responsive, and reconfigurable structures.^1,2^ Underlying this programmability is the sequence-specific hybridization of short DNA strands that facilitates selective interactions between particles coated with complementary sequences.^1,3,4^ Combining the sequence specificity of DNA with other design parameters such as directional interactions,^3,5,6^ particle shape,^7^ surface mobility of the DNA,^8^ and kinetic control over the assembly process, researchers have assembled a range of structures, such as crystals,^9,10^ quasicrystals,^11^ metamaterials,^12^ gels, and lower dimensional structures, such as chains, rings, and stick figures.^13–15^ These studies show that DNA coatings can be used to organize particles with varied chemical make-up into precisely micro-structured materials.^1,16^

The DNA coating on a single microparticle contains tens of thousands of strands,^10,17–19^ and in principle has the information capacity to encode on the order of 100 orthogonal interactions with potential binding partners after accounting for geometric, entropic, and sequence overlap effects.^20^ The ability to encode such a large number of selective and orthogonal interactions on a single building block, and the large self-assembly design space it offers, is the central promise of DNA-mediated assembly.^21,22^ Indeed, simulations showed that mixtures of such multi-specific (also called promiscuous or polygamous^20^) particles can assemble into complex and finite-sized structures^23^ and are key to making clusters with dynamic properties such as shape shifting, catalysis, and self-replication.^24,25^

While experimental studies demonstrated the possibility of encoding multiple interactions on a single particle^6,13,20,26,27^ and opened the door to multifarious self-assembly,^28^ a gap remains between the number of distinct binding partners successfully implemented in experiments and those envisioned in simulations. Experiments showed that, even with few sequences per particle, the competition between intra- and inter-particle DNA bond formation can result in interesting behaviors, such as binding only after prolonged contact,^13,29^ re-entrant melting,^30^ or reconfiguration of crystal structures.^6^ Simulations on the other hand showed that increasing the number of orthogonal interactions per particle makes it possible to self-assemble finite-sized, aperiodic architectures.^23–25^

Orthogonal interactions alone are not sufficient to realize the structural complexity seen in simulations. Equilibrium assembly of DNA-coated colloids requires the interaction strengths of all binding partners to be tuned such that every interaction is reversible at the same temperature. This is a difficult task because the temperature window for reversible interactions between DNA-coated colloids is notoriously narrow – on the order of 1 °C^10,20,31,32^ – and depends not only on the individual binding energy of each sequence but also on the number of strands of each sequence in the DNA brush. For successful equilibrium assembly of multispecific particles, these temperatures must be synchronized across all sequences involved, a condition that demands strategies for fabricating colloids with mixed DNA coatings whose compositions can be precisely controlled.

Here, we show how the DNA sequence composition of multispecific colloids can be tuned and how closely the resulting coatings match their intended compositions. We compared two strategies: (i) simultaneous grafting of mixtures of functionalized DNA sequences, whose composition is often assumed to reflect the input ratio,^6,10,13,19,20,26,33,34^ but which is rarely verified,^19,34^ and (ii) appending new binding domains onto pre-grafted strands by isothermal DNA polymerization.^33,35^ We then determine how coating composition influences assembly pathways and structures. Together, these results reveal the conditions under which multispecific particles assemble simultaneously and reversibly, rather than sequentially by diffusion-limited aggregation, thereby establishing key parameters that govern the self-assembly of complex DNA-programmed colloidal materials.

## 2 Results

### 2.1 Coating composition control with DBCO-click chemistry

**Fig. 1:**
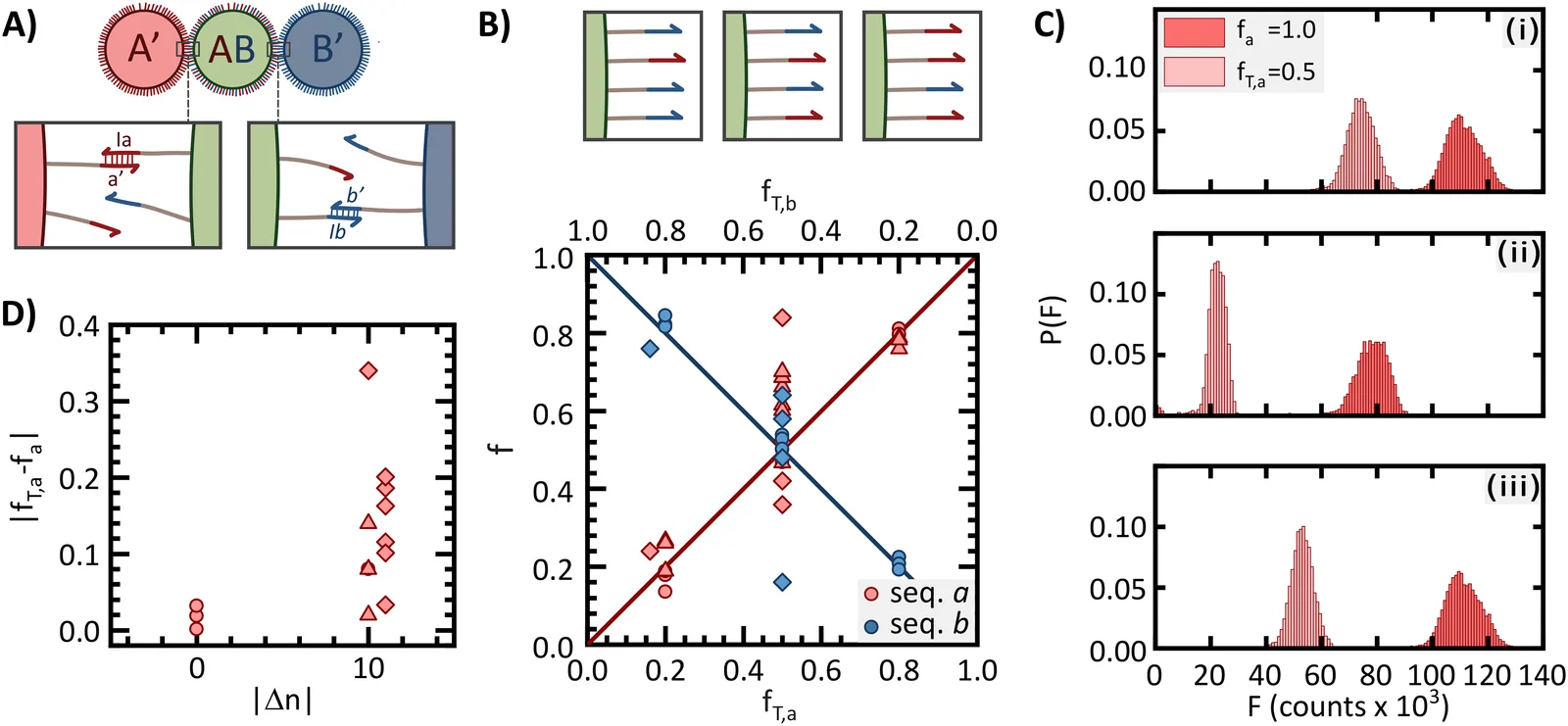
(A) Schematic representation of multispecific interactions. Particles of type AB, functionalized with two distinct DNA sequences, can simultaneously bind to complementary A′ and B′ particles. On these multispecific particles, the relative grafting densities of the two DNA sequences can be tuned. (B) Dual grafting of distinct DNA sequences onto single particles *via* DBCO-click chemistry was achieved by mixing different ratios of DBCO-functionalized DNA strands. The graph compares the target (input mixing) ratios *f*_T,a_ and *f*_T,b_ with the measured grafting fractions *f* for three different sequence combinations. In all cases, sequence a (red) was kept the same and paired with (i) a poly-thymine sequence lacking a sticky end (triangles), (ii) a sequence b of the same length (circles), or (iii) an extended version of b (rhombus). The data show good agreement between the target and measured grafting at low and high target ratios, with increased variability observed near equal grafting fractions. (C) Probability histograms of the fluorescent signal *F* measured for 10 000 individual particles. Data of sequence a are shown for each sequence combination given in panel B, all targeting a fraction *f*_T,a_ = 0.5, and compared to reference particles with *f*_a_ = 1 (fully grafted with sequence a). Here, the fluorescence intensity reflects the extent of grafting, while the width of the histograms reflects the degree of particle-to-particle variation in grafting density. (D) Deviation of the measured grafting *f*_a_ from the target *f*_T,a_ = 0.5 *versus* the nucleotide length difference |Δ*n*| between sequence a and its partner.

A straightforward way to prepare multispecific DNA-coated colloids (*i.e.* particles with multiple sequences in their coating, Fig. 1A) is to mix functionalized DNA strands and graft the mixture onto particles. If the grafting reaction is independent of the sequence and local chemical environment, the composition of the mixture should directly translate into the composition of the coating. This strategy is used not only to produce multispecific particles,^13,20,26,33,36^ but also to tune the grafting density of a single sequence by mixing it with an inert strand such as poly-T.^10,19,20,26,34^ Depending on the surface chemistry, the DNA may be biotin-labeled and react with streptavidin-coated surfaces, or DBCO-labeled and react with azide-functionalized particles, but in either case, it is unclear whether the assumption that the DNA mixture determines the DNA coating composition holds. Here, we focus on DBCO-labeled DNA, which is known to yield dense, homogeneous coatings suitable for self-assembly.^17^

To measure how well the composition of the DNA coating on the particles' surfaces reflects the mixing ratio of DNA strands in the reaction mixture, we grafted particles using the mixed-DNA click method for a single functional sequence, a, mixed with an inert poly-T strand, quantified the fraction of surface grafted DNA with sequence a, *f*_a_, and compared the obtained ratio with the target ratio. We targeted grafting fractions, *f*_T,a_, of 0.2, 0.5 and 0.8, using a total DNA density on the surface of the particle of *ρ*_max_ ≈ 10^5^ strands per particle (SI section 3). In order to quantify the grafting density, we hybridized the particles with complementary DNA strands with a fluorescent group and measured the fluorescent signal per particle using flow cytometry (see Methods).

Fig. 1B shows the measured fractions as a function of the target composition for three independent syntheses (triangles). While the average measured fraction agrees well with the target composition (SI section 2), we observe substantial variation between data points, particularly at a target ratio of 0.5. This indicates that grafting a mixture of two DNA strands in a 1 : 1 ratio onto the particle surface is not guaranteed to produce particles with the same DNA ratio in their DNA brush.

To investigate the origin of this variation, we first looked at the particle-to-particle variation (*i.e.* how the composition of DNA brushes varies between particles in one sample) using the particle's fluorescence intensity as a proxy for the number of strands in their coating. We found that the distribution of fluorescence values was comparable in width for reference particles with pure DNA coatings and mixed-coating particles (Fig. 1C(i)). This indicates that the broad spread observed in Fig. 1B arises primarily from batch-to-batch variation (*i.e.* differences between independently prepared samples), rather than from particle-to-particle variation within a single sample.

Next, we asked whether the length of the DNA strands affects the coating composition and found that the degree of batch-to-batch variation depended on the length difference between the two DNA strands (Fig. 1B and D). The spread around the target composition was large when mixing a strand a with a shorter poly-T sequence (triangles), but mixing two strands of comparable length, binding energy, and nucleotide composition produced coating compositions that closely matched the expected trend (circles). Mixing sequence a with an 11-nucleotide longer DNA sequence b (rhombus) again resulted in variable grafting fractions, similar to what we found for mixing sequence a with an inert poly-T sequence. Surprisingly, a length mismatch did not cause one of the strands to be systematically overrepresented on the particle surface, which would be expected if the adsorption efficiency or reactivity of the strands depended on their length – such dependence was reported for similar click reactions on silica nanoparticles,^37^ and we indeed found that the overall grafting density depends on the sequence (SI section 3). These findings suggest that the spread around the target coating composition comes from a stochastic effect on the scale of the sample that is somehow more prevalent for strands of different sizes than for strands of the same size, but we do not understand by what mechanism.

Together, our results show that the mixing ratio of DNA strands in the solution cannot be taken as a proxy for the DNA in the coating on the particle and present a need for alternatives to the mixed-DNA click method to engineer multicomponent DNA coatings with precisely tunable mixing ratios for fully addressable self-assembly.

### 2.2 Coating composition control with the primer-exchange reaction

**Fig. 2:**
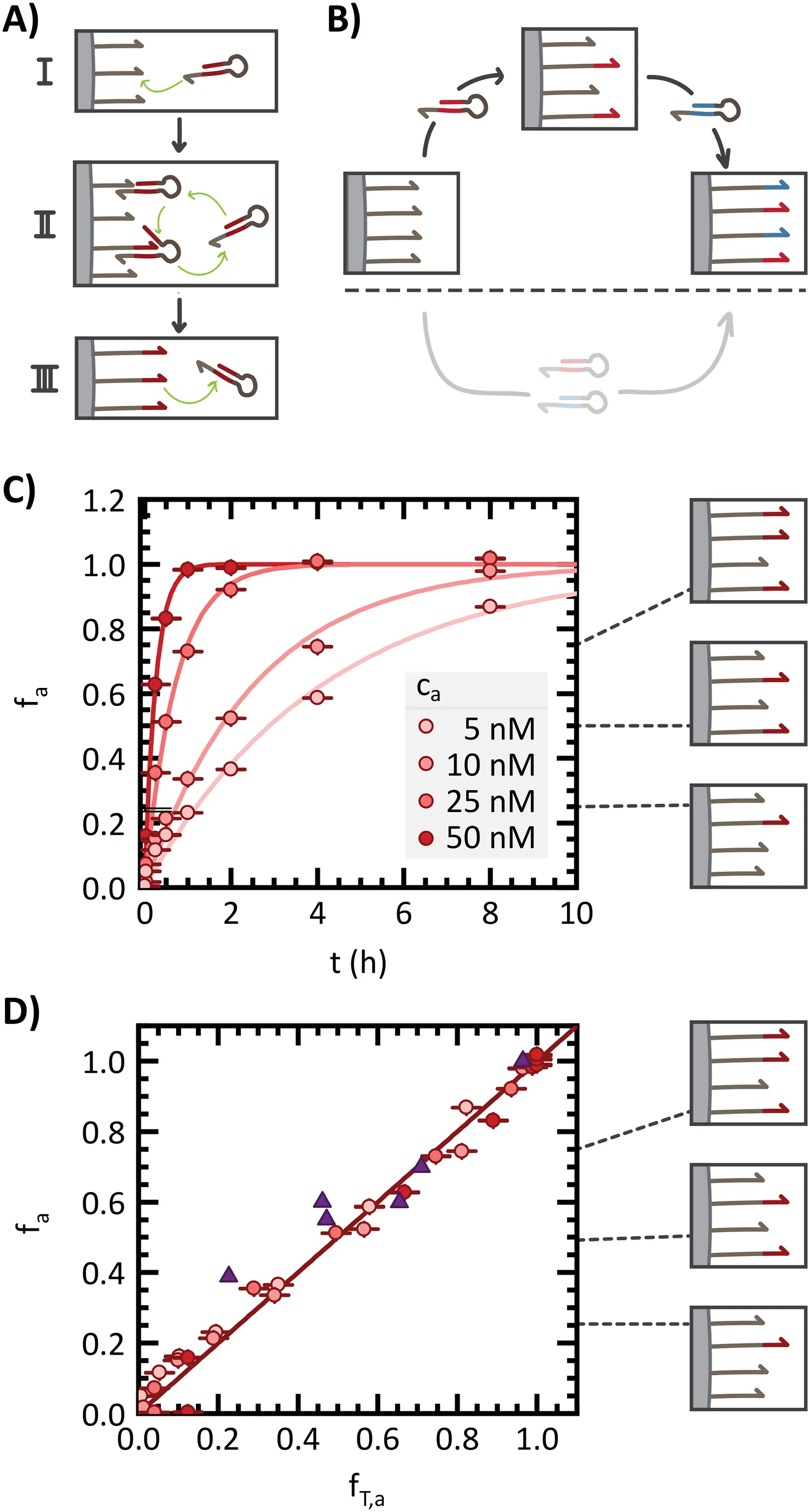
(A) Primer exchange reaction (PER) mechanism. (I) A DNA template hybridizes to the particle-grafted strand. (II) The strand is extended by template-directed isothermal DNA polymerization. The template's hairpin enables reversible binding, allowing reuse on the next strand. (III) The reaction ends once all input strands are extended. (B) Multispecific particles produced by the multi-step PER. In step one, a sequence is grown and the reaction stopped early, leaving unreacted sites. In step two, these sites are extended with a different sequence *via* template exchange. (C) The grafted fraction of new DNA domains *f*_a_ grows exponentially over time, increasing faster at higher template concentrations *c*_a_. (D) Data from panel C replotted against the expected fractional conversion *f*_T,a_. Expected fractions were calculated from the reaction time, template concentration, and the mean reaction rate from three measurements. Purple triangles show independent reaction batches, distinct from panel C, run to reach specific target fractions. Error bars in all panels represent the standard error of the mean.

For more precise control over the composition of mixed DNA coatings, we explored an alternative approach based on the primer exchange reaction (PER), an isothermal DNA polymerization reaction.^38^ The PER enables the addition of new DNA domains onto particle-grafted DNA using a DNA template in a hairpin structure as a catalyst.^35^ The template binds reversibly to the grafted DNA (primer), and in the presence of nucleotides and polymerase, its double-stranded region is copied, and the new domain is covalently attached to the primer. The hairpin then detaches and can catalyze another reaction (Fig. 2A).

The PER rate can be precisely controlled by adjusting the template concentration (SI section 6),^35,38^ so we asked whether we could create a DNA coating with tunable composition by halting the reaction before it reaches completion. In the context of multispecific coatings, halting the initial reaction before completion leaves unreacted sites that can be targeted with a different DNA sequence in a subsequent functionalization step (Fig. 2B). Alternatively, multispecific coatings could be prepared through multiple simultaneous competing primer exchange reactions. Then the ratio of template concentrations would control the composition of the coating.

#### 2.2.1 Multi-step PER

Control over DNA coating composition in the multi-step PER depends on the precise control of completion in the initial step, which is governed by reaction time and rate. To target a specific completion in the first step, we measured the rates with which new DNA domains are grown on the particle as a function of the PER template concentration. To quantify these rates, we quenched the PER at various time points, added fluorescent DNA complementary to the newly polymerized domains, and measured the fluorescent signal as a function of the reaction time in triplicate (SI sections 5.1 and 5.2). The results of these measurements are shown in Fig. 2C. Consistent with earlier work,^35,38^ we found that the fraction of grafted DNA strands that contain the newly polymerized domains follows first order reaction kinetics and can be described by *f*_a_ = 1 − e^−*k*_a_*t*^, where *f*_a_ represents the measured fraction of the DNA grafted onto the colloids onto which we grew a new functional domain a: *f*_a_ = *ρ*_a_/*ρ*_0_, *t* is the reaction time, *ρ*_0_ is the total DNA grafting density measured from the plateau (SI 5.2), and *k*_a_ is the template-concentration-dependent reaction rate.

To assess the level of control that this method could give over the grafting density, we calculated an expected fractional conversion based on the reaction time *f*_T,a_, the average reaction rate obtained from the fits, and the template concentration. We plotted the data from Fig. 2C on this rescaled *x*-axis in Fig. 2D. The plot shows a small deviation of each individual data point from the expected fractional conversion calculated from the average reaction rate, indicating that each reaction batch closely matched the conversion predicted from the model and suggesting that the PER could give precise control over the composition with little batch-to-batch variation.

Next, to independently test to what extent the target grafting density can be obtained, we chose template concentrations and reaction times to target a series of specific grafting fractions, 0.23, 0.47, 0.65, 0.71, and 0.96, and plotted the results in Fig. 2D (purple triangles). Since the reaction was not completed in these experiments, the total number of DNA strands on the particle (*ρ*_0_) cannot be obtained from the plateau. Instead, we define the fractional conversion as *f*_a_ = *ρ*_a_/*ρ*_ref_, where *ρ*_ref_ is the grafting density measured on a separate reference sample that was fully extended to sequence a under saturating PER conditions.

Fig. 2D shows that the independent data points closely match the target line, typically deviating by less than 10%. These deviations are somewhat larger than those observed in the original datasets (red circles), which were used to determine the average *k*_a_ values and target densities. This difference is likely due to the variation in the time at which the reaction was stopped. Particularly, at higher template concentrations and short reaction times, the reactions are faster and small variations in timing can substantially impact the final grafting fraction. Consistent with this notion, the variation was larger for smaller target conversions.

Building on these results, we return to multispecific particles, which are generated by extending unconverted DNA strands with a second domain b in a second reaction step. To test this approach, we grew a second domain b onto the particles with *f*_a_ = 0.55 from Fig. 2D, and measured the grafting density of both sequences using flow cytometry (SI section 8). After completion of the second step, domain b was added to a fraction *f*_b_ = 0.31, resulting in *f*_total_ = 0.86. Curiously, the total of the two sequences did not add up to 1. This mismatch could be due to variability in the final grafting densities observed between PERs, even when they are initiated from the same particle batch (SI section 5.2), or to the possibility that the grafting density of our reference particles is higher than that of the particles tested here (*ρ*_0_ < *ρ*_ref_). Although this mismatch did not pose an issue for grafting a single sequence at a controlled density *ρ*_a_, it limits control over the second sequence *ρ*_b_, which was grafted at a substantially lower density than intended.

The uncertainty about the total grafting density poses a problem for making particles with many different sequences. As the number of unique grafted sequences increases, the number of required reaction steps increases accordingly, and the error is expected to accumulate. Each subsequent reaction step requires a reaction time that depends not only on the intrinsic reaction rate but also on the number of unreacted binding strands, which is in turn influenced by the previous grafting steps. Consequently, the spread in the coating composition is expected to grow as additional unique sequences are added.

Together, our results show that the sequential PER method provides good control over the grafting density of a single sequence, but is less suitable for multispecific DNA coatings. To sidestep issues associated with variations in *ρ*_total_ and the propagation of errors in multiple PER steps, we next asked whether multispecific particles can be prepared by adding multiple sequences to a DNA coating simultaneously rather than in a stepwise manner.

#### 2.2.2 Competitive PER

**Fig. 3:**
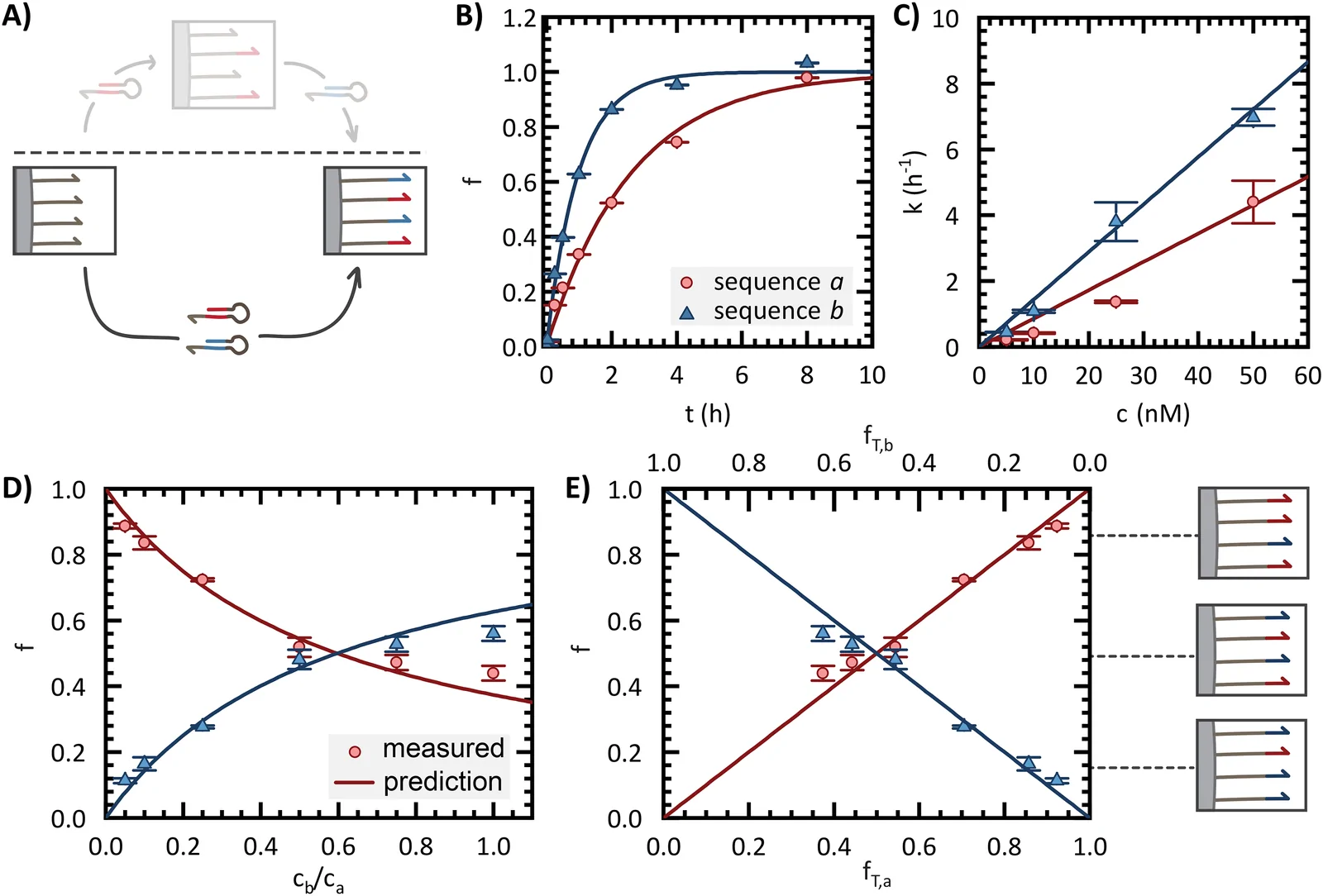
(A) Competitive PER using a multi-template design, where two different DNA domains are grown simultaneously on the same particle. (B) Grafting fractions *f* of each sequence increase exponentially with reaction time, with sequence a reacting more slowly than sequence b. (C) Reaction rates of the PER *k* scale linearly with template concentration c, allowing control through template input. (D) In the competitive PER, the grafting fraction of each sequence is tuned by the relative template concentrations *c*_a_ and *c*_b_ that control the growth rate of sequences a and b, respectively. The *x*-axis represents the ratio of the two templates, with a fixed *c*_a_ = 10 nM and varying 0.5 nM < *c*_b_ < 10 nM, such that 0 < *c*_b_/*c*_a_ < 1.0. For each particle, the grafting fractions *f*_a_ and *f*_b_ are measured independently, producing paired data points that are compared to the corresponding target compositions *f*_T,a_ and *f*_T,b_. These targets are predicted fractions based on rates from panel C and eqn (1) and are indicated by the solid lines. The data points closely follow the predicted trend. (E) Measured grafting fractions *f* for both sequences agree well with the target graftings for sequence a *f*_T,a_ and sequence b *f*_T,b_ (solid lines), showing only minor deviations. Error bars in all panels represent the standard error of the mean.

Adding multiple sequences to a DNA coating in a single step requires the PER with multiple templates that compete for primers on the particles' surfaces (Fig. 3A). In this approach, the composition of the DNA coating is set by the relative reaction rates with which the domains are grafted onto the surface. The rates can be controlled with the relative concentrations of the templates, but also depend on the sequence of the DNA domain that is added.^38^

To produce particles with well-defined ratios of two sequences in their DNA coatings, we first measured the inherent PER rate for both sequences, *k*_a_ and *k*_b_. Fig. 3B shows *f*_a_ and *f*_b_ as a function of reaction time at a template concentration of *c*_a_ = *c*_b_ = 10 nM. It shows that sequence b grows roughly twice as fast as sequence a, despite the fact that the binding domain is the same for both templates (gray in Fig. 3A). We found that this observation holds for a range of template concentrations (SI section 7.1).

Such sequence dependence is consistent with earlier observations that growth rates depend strongly on the sequence of the domain that is polymerized.^38–40^ These rate differences are therefore inherent to using multiple distinct sequences and cannot be eliminated by simple design choices. The ratio of template concentrations alone is therefore not sufficient to control the composition of the DNA coating; intrinsic sequence-dependent differences in polymerization rates must be corrected.

To correct for sequence-dependent differences in the PER rate, we first measured the PER rate of both sequences as a function of the template concentration. Fig. 3C shows that the PER rate increases linearly with template concentration for both sequences a and b, but with different sequence-dependent slopes, 
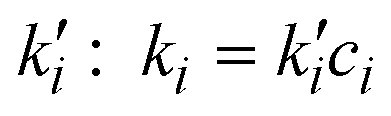
. This finding suggests that, although different sequences grow at different absolute rates, their relative growth can still be controlled by adjusting template concentrations. To correct for the sequence dependence, we assume that both primer exchange reactions proceed independently and express the fraction of the DNA on the colloid that turns into sequence *i* as a function of both the inherent rates *k*_*i*_ and *k*_*j*_ and the template concentrations *c*_*i*_ and *c*_*j*_:1
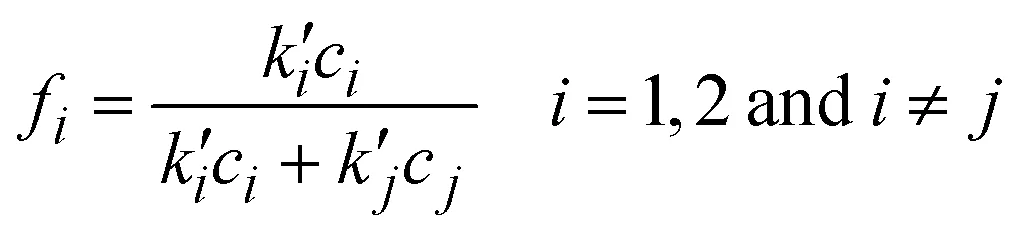


Eqn (1) gives the relative concentrations required to reach a target composition of the DNA coating *f*_*i*_ and *f*_*j*_ = 1 − *f*_*i*_. To test whether we can prepare multispecific particles with a target DNA composition, we mixed templates of sequences a and b, keeping the template concentration of sequence a constant at *c*_a_ = 10 nM and varying the template concentration of sequence b between 0.5 nM and 10 nM. We compared the measured DNA composition with the prediction from eqn (1) in Fig. 3D and found good agreement between the experimentally measured grafting fractions and the prediction from eqn (1), with deviations of up to 10% from the target. This result shows that a sequence-dependent competitive disadvantage can be compensated with increased template concentration.

The fact that rate-corrected template concentrations yield grafting ratios close to the targets shows that the two PERs behave effectively independently, despite competing for the same input strand. This independence suggests that template binding competition does not affect the relative reaction rates in the concentration range used here. We found that the simple proportionality breaks down, as expected, at high template concentrations, where the binding between the template and the primer becomes the rate limiting step (SI section 7.1). Hence, we kept the sum of the template concentrations well below this limit (≈50 nM).

To directly compare the mixed click approach with the competitive PER approach for producing particles with DNA coatings of specific compositions, we rescaled the *x*-axis of Fig. 3D with eqn (1) to represent the intended grafting fraction (Fig. 3E). This graph can be directly compared to Fig. 1B and shows that the spread around the targeted grafting fractions is consistently smaller for the competitive PER than for the mixed click approach.

Taken together, we found that, with the competitive PER, intended coating compositions can be produced consistently across a wide range of compositions with a precision of a few percent, but this requires a systematic correction for the inherent rate differences between sequences through the adjustment of the relative template concentrations during the reaction.

### 2.3 Tuning the interaction strength of DNA-coated colloids for equilibrium assembly

Having established the precision with which the composition of DNA coatings can currently be controlled, we next ask what the consequences are for the self-assembly of multi-specific particles. How many independent DNA-mediated interactions that are reversible in the same temperature window can we reasonably engineer between one type of particle and other particles in the mixture?

**Fig. 4:**
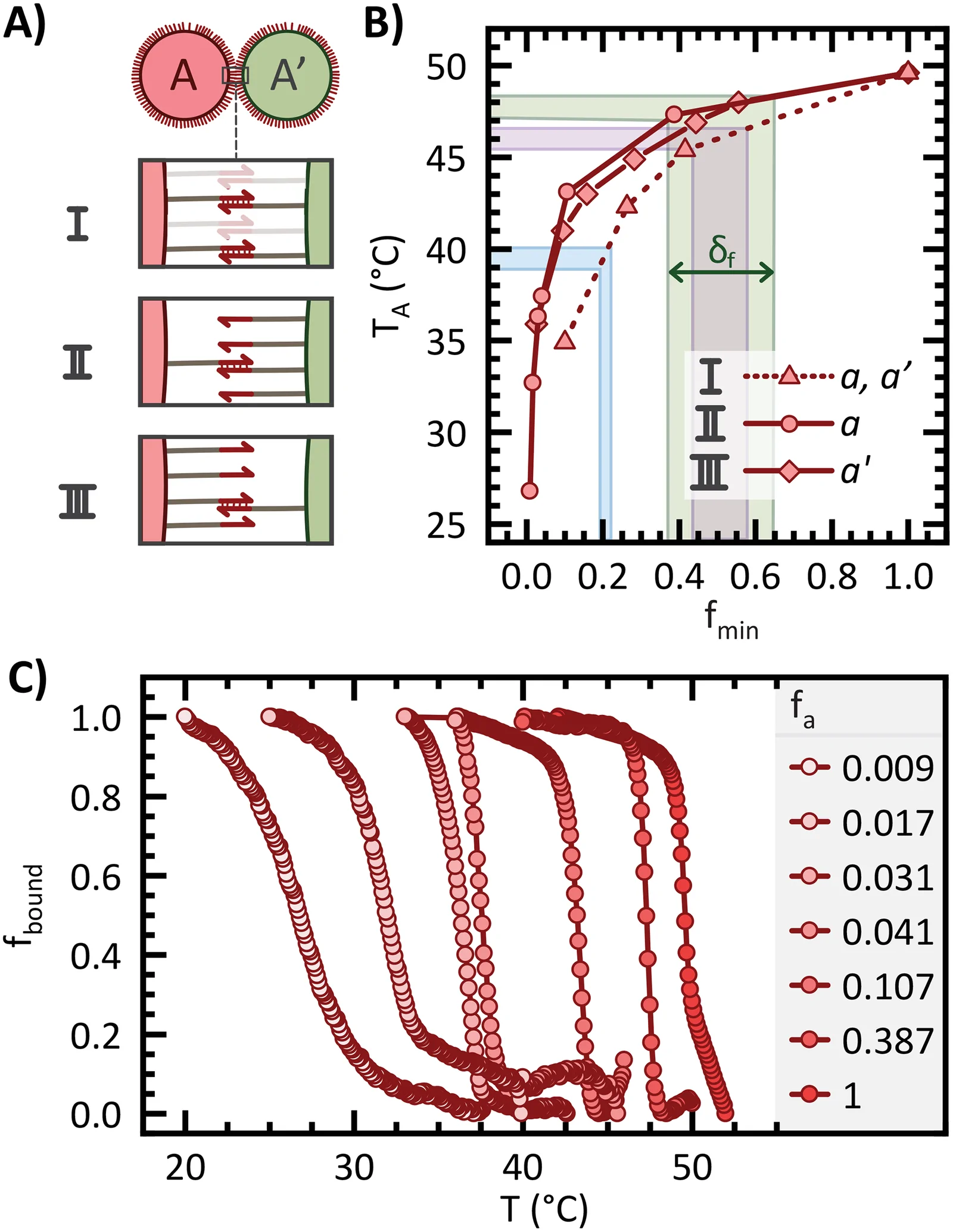
(A) Schematic representation of the binary system pf particles coated with either sequence a (red) or its complementary sequence a′ (green), where grafting densities are reduced either on (I) both binding partners equally or (II and III) on only one of the partners. (B) Measured assembly temperatures *T*_A_, the temperature where particles reversibly assembly in clusters, plotted as a function of the lowest grafting fraction *f*_min_ of the two particle types (with grafting fractions *f*_a_ and *f*_a′_), where *f*_min_ denotes the smaller of the two. Assembly temperatures decrease steeply with lower grafting densities. For system I, this decline occurs at higher overall grafting densities, showing that the assembly temperature is determined not only by the partner with the lowest grafting density but also by the combined grafting on both particles. Shaded regions indicate the acceptable range of grafting densities *δ*_*f*_ yielding melting temperatures within 1 °C of the target value. The shading illustrates that this acceptable range narrows as the target fraction *f*_a_ decreases (blue *versus* purple shading), and that the range is wider when the binding partner carries a higher overall grafting density (green *versus* purple shading). (C) Fraction of particles bound to another particle *f*_bound_ against the temperature. Samples of different grafting densities are shown for system II (panels A and B). As samples were cooled, a transition from the singlet phase to the bound phase is observed. For lower grafting fractions *f*_a_, this transition shifts to lower temperatures. The assembly temperatures shown in panel B correspond to the temperatures at which *f*_bound_ = 0.5.

The first step to answering this question is to know how the melting temperature depends on the grafting density. Previous work showed that the melting temperature of DNA-coated colloids depends sub-linearly on the grafting density when the grafting densities of both binding partners are equal^13,20,34^ and that the temperature window in which the interactions are reversible is small, typically 1 °C.^10,20,31,32,41^ We found the same sublinear trend for particles with partial but equal coverage of DNA with sticky ends a and a′, produced with the sequential PER method (triangles in Fig. 4B).

In systems of multi-specific particles, the surface densities of sticky ends of two binding partners need not be equal though, which may affect the melting temperature associated with that DNA-mediated interaction. To study the effect of unequal grafting densities on the melting temperature, we varied only the coverage on the particles with sequence a and measured the melting temperature of these particles mixed with the particles with the maximum grafting density of the sequence a′ (circles in Fig. 4B), and *vice versa* (diamonds in Fig. 4B). Fig. 4B shows the measured assembly temperature as a function of the lowest grafting density of both partners. Both measurements with unequal DNA grafting densities followed the same qualitative trend as those with equal grafting densities, and the temperature range over which the interaction remained reversible was similarly narrow (Fig. 4C). However, the melting temperatures increased more steeply at low grafting densities and transitioned earlier to flattened, sub-linear dependence, converging to the same melting temperatures.

How do we interpret this result? One might assume that the interaction strength between two DNA-coated colloids – and by extension the melting temperature – is determined by the minimum number of DNA connections that can form, and so by the lowest number of sticky ends between the two particles. Then the grafting density of the more densely coated particle should not matter, but Fig. 4B shows that it does. The difference between the curves with equal and unequal grafting densities in Fig. 4B is a consequence of the entropic contribution of binding between DNA-coated colloids; each strand on the lower density particle has multiple possible connection points on the other higher-density particle, which stabilizes the interaction.^13,34^

The consequence of this effect for multi-specific particles is that the melting temperature is determined not only by the composition of the multi-specific particle in question but also by the composition of its binding partner. This means that greater flexibility in DNA composition is available when targeting a specific melting temperature if the binding partner is fully coated with a single DNA type, rather than being multi-specific.

Consider this numerical example to see the consequence of this effect. A particle with two types of DNA (each with *f*_*i*_ = 0.5) in its coating has an acceptable range *δ*_*f*_ of 10% in grafting density to still be within the 1 °C window around the target melting temperature when it's binding partner has a full coating of the complementary DNA (green shading in Fig. 4B). When the binding partner also has two DNA types with each a fraction of 0.5, the acceptable range is only 5% (purple shading).

The constraints on coating composition become more stringent as more sequences are added to the coating because each additional sequence pushes the system closer to the steep regime of the melting curve. For example, if the particle in question has 5 different types of DNA in its coating with each a fraction of *f* = 0.2, the acceptable variation in composition is only 0.5% even when each binding partner carries only one type of DNA (blue shading). This constraint effectively limits practical implementation to two or three distinct sequences per particle.

We conclude that, although many orthogonal interactions could in principle be grafted on a single particle, for the purpose of equilibrium self-assembly we are much more limited; for DNA-coated colloids with grafting densities of 10^5^ strands per μm^2^, and given the precision with which DNA coatings can be controlled, the number of reversible orthogonal interactions that can be engineered on one DNA-coated colloid is limited to two or three and only if these interactions are with mono-specific binding partners. This estimate assumes that each DNA strand interacts independently with no intra-particle interactions and that each sequence has the same free energy of association. The realizations of the equilibrium programmable, addressable assembly of many colloidal building blocks shown in simulations^23–25^ thus require a substantial improvement in the maximum DNA density that can be obtained, the precision with which the coating composition can be controlled, or a widening of the 1 °C temperature window for equilibrium assembly, or fine-tuning methods to the melting temperature associated with each interaction through the addition of competitor or linker strands.^30,42–45^

### 2.4 Equillibrium assembly of multispecific DNA-coated colloids

Having shown that equilibrium assembly is practically limited to systems in which the relevant sequences can share a narrow assembly-temperature window, typically on the order of two or three DNA sequences on a particle, we now test explicitly whether our grafting-density control is sufficient to achieve such three-component assembly. To test this, we prepared a system of three particle types: one multi-specific, coated with sequences a and b and two mono-specific: one coated with only a′, and one with only b′. We varied the composition of the coating on the multi-specific particles and tested whether we could identify the regime in which all components co-assemble into a single, ordered structure. For the assembly experiments, we followed the typical annealing protocol required for crystallization:^10,18,41,46^ heat to above the melting temperature and cool slowly with a rate below 0.3 °C hour^−1^.

**Fig. 5:**
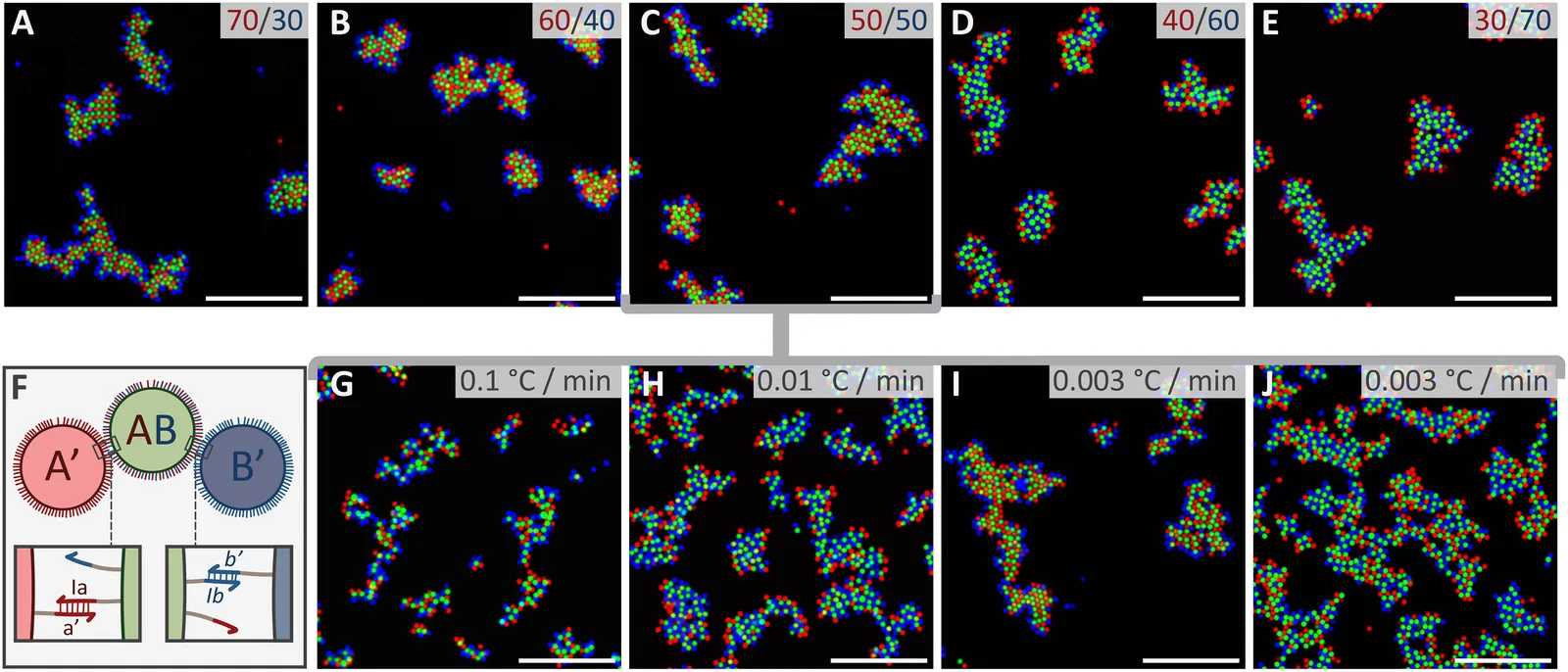
Various self-assembled structures of multispecific particles (green) with their complementary binding partners (red and blue). The scalebar is 20 µm. (A)–(E) Structures formed for different grafting fractions of the two sequences on the multispecific particle during a cooling ramp of 0.003 °C min^−1^. Core–shell structures are observed that result from stepwise assembly, with the order of assembly switching depending on the grafting fractions. (F) Schematic illustration of the interactions between the respective particles. (G)–(J) Structures formed from 50/50 multispecific particles and their complements under different cooling ramps. Panels (G)–(I) show that the resulting structures depend on the cooling rate, with the order of assembly varying between rates. Panels (I) and (J) highlight that independent duplicates under the same cooling ramp can still yield different structures due to unavoidable sample and temperature variations.

We found that during thermal annealing the particles typically assembled stepwise (SI section 11.2), resulting in the formation of core–shell structures (Fig. 5A–E). Such stepwise assembly is expected when the binding energies between the multi-specific particles and their binding partners – and by extension the melting temperatures – are unequal.^20,26^ In this case, the multi-specific particles first form dense clusters with one binding partner, and at lower temperatures, the second type of binding partner attaches to the previously formed clusters. Indeed the samples with *f*_a_ = 0.7 and *f*_a_ = 0.6 formed binary crystals in which the particles coated with a′ are surrounded by particles coated with b′ (Fig. 5A and B). The opposite core–shell structure formed when the multi-specific particles had the inverse composition *f*_a_ = 0.3 and *f*_a_ = 0.4. (Fig. 5D and E).

Particles with equal grafting density of both strands (*f*_a_ = 0.5, *f*_b_ = 0.5) still formed core–shell structures with the a′ coated particles in the core (Fig. 5C). This asymmetry can be attributed to a small difference in free energy of hybridization between the two sequences (−13.98 kcal mol^−1^ for sequence a *vs.* −12.94 kcal mol^−1^ for sequence b) for single strands free in solution, such that equal binding strengths are expected at a grafting ratio of 48/52 rather than 50/50 (SI section 9).

The difference between the target 50/50 DNA ratio and the composition of 48/52 that we predict to equalize the two binding strengths is smaller than the precision with which we can control the composition. Furthermore, this difference should result in a melting temperature difference well within the 1 °C assembly window indicated in Fig. 4, and yet we find that these particles did not co-crystallize under the applied thermal annealing protocol. Therefore, we asked next how the applied temperature protocol affected the assembly. We tested three different cooling rates (0.1 °C min^−1^, 0.01 °C min^−1^ and 0.003 °C min^−1^) and observed markedly different structures for each. At relatively fast temperature ramps, mixed and disordered clusters formed (Fig. 5G), whereas slower ramps produced demixed structures in which crystalline domains of two particle types are surrounded by a shell of the third type. This contrast indicates a tradeoff in the annealing rate that complicates equilibrium assembly of multi-specific particles: slower cooling promotes ordered structures, because it facilitates reversible binding and local reorganizations; but it also hinders co-assembly because it makes the system more sensitive to small differences in melting temperatures and the associated differences in nucleation rates (Fig. 5G and H).

The comparison between the structures formed at various annealing rates also shows how sensitive the system is to such small differences in the nucleation rate. Fig. 5H and I show the same sample cooled at 0.01 °C min^−1^ and 0.003 °C min^−1^. Both produced core–shell structures but with opposite core- and shell-compositions. At a cooling rate of 0.003 °C min^−1^, two independent duplicate samples were measured (Fig. 5I and J). In one sample, core–shell clusters formed while in the other the three particles co-assembled into a dense mixed structure with local crystalline domains. These results show how minute differences in the particle concentration or in temperature (the heat conductivity of each sample cell might vary slightly) can affect the nucleation rates such that equilibrium co-assembly of even only three types of DNA-coated colloids requires substantial fine-tuning.

What do these findings mean for the experimental realization of simulations of fully addressable assembly? Even with precise control over the coating composition, minute deviations in local particle concentration, temperature, and interaction strength cause particles to tend to assemble in stages rather than simultaneously under a slow thermal annealing protocol. This effect is exacerbated by the collectivity of multivalent binding in systems of DNA-coated microparticles,^34,47^ but even in systems of DNA-coated nanoparticles and DNA origami building blocks the assembly rates strongly depend on temperature and binding strength.^48–50^ Together, this means that equilibrium calculations are insufficient for structure prediction in co-assembly of multiple building blocks and that assembly kinetics need to be considered.^1,26,51^

## 3 Conclusions

Realizing the full potential of DNA-coated particles for programmable self-assembly requires the functionalization of particles with multiple DNA sequences that interact orthogonally.^23–25^ In this work, we showed that the primary limitation on the number of orthogonal equilibrium interactions that can be implemented is not the number of possible orthogonal DNA sequences that can be designed, nor the total number of unique DNA sequences that can be grafted. Instead, it is set by the requirement for equilibrium assembly that all interactions operate in the same narrow (≈1 °C) temperature window in which DNA-mediated bonds are reversible.

Because the binding strength—and consequently the accessible temperature window—of each interaction depends on the surface density of each DNA strand type, variations in the DNA coating composition change the temperature at which each interaction is reversible. We compared a mixed click-chemistry method with a PER-based method for producing particles with mixed DNA coatings and found that both methods resulted in variations of approximately 10% around the target density when the sequences in the coating had the same length. When the two sequences differed in length, the click-based method could produce variations of more than 30% from the target. Even in the best-case scenario of a 10% variation around the target composition, the steep dependence of DNA-mediated interactions on grafting density means that designing particles with more than two types of equilibrium interactions encoded in their brush is not currently feasible.

Going beyond two equilibrium interactions requires strategies to fine-tune interaction strengths during self-assembly. Free-floating DNA strands could be used as linkers or competing strands,^30,42–45^ so that the concentrations of these strands would selectively modify one of the interaction strengths in the system to bring all transition temperatures in the same 1 °C window. Removing the constraint of equilibrium interactions, the sequence space of DNA oligos of 11 nucleotides facilitates around 70 orthogonal interactions between colloidal particles.^20^ This design space could be explored by facilitating local reorganizations using surface-mobile DNA linkers^8,52^ so that particles can reorganize locally even if the DNA-mediated interactions are not near the melting temperature. The property that multispecific particles will almost inevitably assemble sequentially—one interaction type at a time—upon thermal annealing could also be harnessed to drive folding-based, line-like colloidal assembly^53^ or core–shell assembly,^54^ without the need to modify the interactions on the go. Because the inherently pathway-dependent and non-equilibrium nature of such sequential multicomponent assembly precludes structure prediction from equilibrium considerations alone, simulation studies will be essential in establishing the design rules to exploit this behavior.

Beyond self-assembly, control over the composition of multi-specific DNA coatings is important for a range of applications. In biosensing, multispecific coatings could enable the simultaneous detection of multiple targets.^55–57^ More broadly, distinct DNA sequences on a single particle could perform distinct roles—such as localizing binding partners,^57^ initiating enzymatic signal production or amplification,^43,44^ or triggering biological responses^58,59^—each with requirements for controlled grafting density.

## 4 Experimental section

### 4.1 Azide-functionalization of polystyrene particles

The azide-functionalized PS–PEO polymer was synthesized following a modified version of the protocol proposed by Oh *et al.*,^17^ where a detailed procedure is provided. Briefly, PS–PEO (polymer source; PS 3800 g mol^−1^, PEO 6500 g mol^−1^) was first sulfonylated by reacting it with methanesulfonyl chloride in anhydrous dichloromethane and triethylamine under ice-bath conditions, followed by stirring overnight at room temperature. The polymer was then precipitated and washed three times in diethyl ether/methanol to remove residual reagents. In the second step, the sulfonyl-modified PS–PEO was reacted with sodium azide in anhydrous dimethylformamide at 65 °C for 24 h. The resulting azide-modified polymer was again precipitated and washed in diethyl ether/methanol, after which the supernatant was removed and the purified PS–PEO–N_3_ was freeze-dried and stored at −20 °C until use.

Next, polystyrene particles were coated with PS-*b*-PEO–N_3_ as follows. The PS-*b*-PEO–N_3_ polymer was dissolved in Milli-Q water at 4 mg mL^−1^ and sonicated overnight. In a glass vial, 150 µL of polystyrene particles (2.6 wt%, 1.1 µm; PolySciences Polybead Sulfate Microspheres, nominal diameter 1.00 µm) was mixed with 86 µL of the polymer solution. The vials were placed on an orbital shaker for 1 h, after which 125 µL of tetrahydrofuran (THF) was added. The samples were shaken for 1 h with the lids closed, then the lids were removed and shaking was continued for an additional hour. Subsequently, 1 mL of Milli-Q water was added, the vials were closed again, and the samples were left on the shaker overnight. The particles were then washed by centrifugation, the supernatant was replaced with Milli-Q water, and the particles were stored at 4 °C. For confocal microscopy (Fig. 5), particles were fluorescently labeled with bodipy 493, bodipy 633, or rhodamine B to distinguish different species. The dyes were prepared at 2 wt% in toluene and 5 µL of dye solution was added during the polymer-functionalization protocol simultaneously with the THF addition.

### 4.2 DNA coupling to particles using click chemistry

Polystyrene particles were functionalized with single-stranded DNA *via* strain-promoted azide–alkyne click chemistry. A suspension containing 0.1 wt% particles and 3.5 µM DBCO-functionalized DNA (Integrated DNA Technologies; custom HPLC-purified oligonucleotides supplied at 100 µM in TE buffer consisting of 10 mM Tris and 0.1 mM EDTA), supplemented with 1 M NaCl and 0.05 wt% Pluronic F127, was heated to 65 °C and continuously mixed for 24 h to promote the covalent attachment of the DBCO-DNA to the azide-functionalized particles. After the reaction, the particles were washed five times with MilliQ water and stored at 4 °C.

Particles were prepared either with a single DNA sequence, by adding 3.5 µM of one type of DBCO-DNA, or with a mixture of two different sequences at varying ratios while maintaining a total DNA concentration of 3.5 µM. The single-sequence functionalization was used to prepare complementary partners in multispecific assemblies and to generate the input particles for the primer exchange reaction experiments. The two-sequence functionalization was used to produce multispecific DNA coatings. An overview of all sequences used is provided in SI section 1.

### 4.3 Attachment of new DNA groups using primer exchange reactions (PERs)

PERs were performed on DNA-coated particles to grow new DNA domains as follows. A PER premix was first prepared at 2.5 times the target concentration, containing 2.5 times the ThermoPol DNA polymerase buffer (New England Biolabs, supplied at 10× concentration), 31.25 mM MgCl_2_ (New England Biolabs; 100 mM stock solution), and 250 µM each of dATP, dCTP, and dTTP (ThermoFisher, 10 mM stock solutions).

To measure PER rates, a 5 µL reaction mixture was prepared consisting of 0.5 µL of 1 wt% DNA-coated particles (final concentration: 0.1 wt%), 2 µL of the premixed PER buffer, 0.5 µL of template solution (concentrations varied between 5 and 100 nM depending on the experiment), and MilliQ water to reach a total volume of 4 µL. The reaction was initiated by adding 1 µL of 0.8 U µL^−1^ Bst large fragment DNA polymerase (New England Biolabs), yielding a final enzyme concentration of 0.16 U µL^−1^. Samples were incubated at 20 °C for 8 hours and aliquots were taken at multiple time points. At each time point, a 0.5 µL aliquot was removed and quenched in 0.1 M EDTA to halt enzymatic activity. Particles were then washed three times with Milli-Q water and stored at 4 °C.

For the preparation of multispecific particles using the multi-step PER, reactions were halted by adding an excess of 0.1 M EDTA solution at defined time points, which were varied to target different DNA grafting densities. For the preparation of multispecific particles using the competitive PER, the same protocol was followed except that two different hairpin solutions were added in varying ratios. The hairpin solutions were premixed before adding the particles to ensure homogeneous DNA binding. Reactions proceeded for 8 h, after which the samples were diluted with 45 µL of 0.1 M EDTA, washed with Milli-Q water, and stored at 4 °C.

### 4.4 Flow cytometry experiments

Flow cytometry was used to quantify DNA grafting densities. Samples were prepared by mixing 5 µL of 0.01 wt% particles with 5 µL of 1 µM fluorescently labeled DNA complementary to the grafted DNA sequence in 40 µL of 1 M NaCl in TE buffer. The mixture was incubated at 4 °C for at least one hour, diluted in 200 µL of the same buffer, vortexed and analyzed using a BD FACS Symphony A3 flow cytometer. Only signals from single particles were collected by applying a gating strategy based on forward and side scattering. Fluorescence was measured for 10 000 particles.

For multispecific particles, complementary fluorescent DNA was added separately for each sequence, and fluorescence was measured independently for each label. To determine the grafting fraction, the fluorescence intensity of multispecific particles was compared to that of two sets of monospecific particles functionalized with the maximum grafting of the corresponding sequences.

### 4.5 Microscopy experiments

Glass capillaries (CM Scientific 0.1 × 2.00 mm) were cleaned by sonicating in isopropanol for 10 min, dried under a nitrogen flow, and treated with plasma for 10 min. The capillaries were then placed vertically in a glass jar containing 200 µL of 1,1,1,3,3,3-hexamethyldisilazane (HMDS) and left to silanize until the solution fully evaporated (approximately 1 day). Afterward, they were stored in acetone. Before use, capillaries were washed with ethanol and dried under a nitrogen flow.

For the measurements shown in Fig. 4, samples were prepared at 0.1 wt% of each particle type in TE buffer containing 250 mM NaCl and 1 wt% Pluronic F127. Samples were loaded into the capillaries, the ends were sealed with grease, and the capillaries were attached to a coverslip (epredia 24 × 60 mm, # 1) using UV-curable glue. A temperature ramp of 0.1 °C min^−1^ was applied using a Linkam heating stage, and samples were imaged simultaneously in bright field using an Olympus BX53M microscope. Assembly temperatures and singlet fractions were extracted from changes in the average image intensity associated with particle cluster formation. Normalized image intensities (0–1) quantified the bound particle fraction *f*_bound_. The assembly temperature (*f*_bound_ = 0.5) was obtained from a sigmoidal fit to the intensity–temperature data.

For the measurements shown in Fig. 5, samples were prepared similarly but with 0.1 wt% multispecific particles and 0.05 wt% of each complementary particle in TE buffer containing 100 mM NaCl and 3 wt% Pluronic F127. Temperature ramps were applied either continuously at 0.1 °C min^−1^ or stepwise, using increments of 0.3 °C or 0.1 °C every 30 min. All ramps were run from 48 °C to 38 °C. The final structures were imaged using a Leica SP8 confocal microscope.

## Author contributions

T. C. M. S. and P. G. M. conceived the experiments. T. C. M. S. and A. S. performed the experiments and analysis. T. C. M. S. and P. G. M. wrote the manuscript. All authors discussed the results and provided feedback on the experiments and manuscript.

## Conflicts of interest

There are no conflicts to declare.

## Supplementary Material

SM-022-D6SM00340K-s001

## Data Availability

All data supporting the findings of this study are provided in the manuscript and the supplementary information (SI). Supplementary information is available. See DOI: https://doi.org/10.1039/d6sm00340k.
