# Supplementary material for: Multispecific DNA coatings for self-assembly
Source: Soft Matter. 2026 Jun 30. Online ahead of print. doi: 10.1039/d6sm00340k (PMC13422231; doi:10.1039/d6sm00340k)
Supplement: SM-OLF-D6SM00340K-s001 [file SM-OLF-D6SM00340K-s001.pdf]

This journal is © The Royal Society of Chemistry 2026

# Supplementary Information

## 1 DNA sequences

| Name | Sequence (5'-3') | Figures |
|---|---|---|
| a | /5DBCON/ 40T ACCATCCTACC | 1 |
| polyT | /5DBCON/ 40T | 1 |
| b (short) | /5DBCON/ 40T ACTCATCTCAA | 1 |
| b (long) | /5DBCON/ 40T C TCCTAATTC ACTCATCTCAA | 1 |
| I | /5DBCON/ 40T ACTTCACTT | 2,3,4 |
| template a | ACCATCCTACC GGGCCTTTTGGCCC GGTAGGATGGT AAGTGAAGT /3InvdT/ | 2,3 |
| template b | ACTCATCTCAA GGGCCTTTTGGCCC TTGAGATGAGT GAATTAGGA /3InvdT/ | 3 |
| Ia | /5DBCON/ 40T ACTTCACTT ACCATCCTACC | 2,3,4,5 |
| Ib | /5DBCON/ 40T ACTTCACTT ACTCATCTCAA | 3 |
| Ia' | /5DBCON/ 40T ACTTCACTT GGATGGT | 4,5 |
| a' | /5DBCON/ 40T GGATGGT | 5 |
| b' | /5DBCON/ 40T TTGAGATG | 5 |
| imager a | GGT AGG ATG GTA A/36-FAM/ | |
| imager b | /56-FAM/TT GAG ATG AGT | |

Table 1 Oligonucleotide sequences used in the study. "40T" indicates a stretch of 40 thymidines. The table also indicates which sequences were used in each figure.

## 2 Statistical relevance of DBCO click chemistry data

| **Sequence combination** | **replicates** | **target a** | **target b** | **mean $\pm$ std a** | **mean $\pm$ std b** |
|---|---|---|---|---|---|
| a + polyT | 3 | 0.2 | 0.8 | $0.24 \pm 0.04$ | - |
| a + polyT | 6 | 0.5 | 0.5 | $0.63 \pm 0.09$ | - |
| a + polyT | 3 | 0.8 | 0.2 | $0.78 \pm 0.02$ | - |
| a + b (short) | 3 | 0.2 | 0.8 | $0.17 \pm 0.03$ | $0.83 \pm 0.01$ |
| a + b (short) | 3 | 0.5 | 0.5 | $0.48 \pm 0.02$ | $0.52 \pm 0.02$ |
| a + b (short) | 3 | 0.8 | 0.2 | $0.801 \pm 0.009$ | $0.21 \pm 0.02$ |
| a + b (long) | 5 | 0.5 | 0.5 | $0.5 \pm 0.2$ | $0.5 \pm 0.2$ |

Table 2 Summary of batch-to-batch variation in the click chemistry experiments, reported as mean $\pm$ standard deviation for each condition. The number of independent replicates is indicated for each entry. This table is provided to complement Figure 1B by supplying statistical descriptors of the data, while the main figure emphasizes the presence of outliers through visualization of individual data points.

## 3 DNA grafting density of monospecific colloids prepared by DBCO-click chemistry

We measured the maximum grafting density $\rho_{total}$ for several sequences using a titration experiment. Particles coated via DBCO-click chemistry (under saturation conditions to achieve maximum coating density) were mixed and left to hybridize with fluorescently labeled complementary DNA strands. Samples were prepared with increasing amount of fluorescent DNA. The fluorescent intensity was measured by flow cytometry and averaged over 10.000 particles. The results are shown in Fig. 1. We observed a linear increase of the fluorescent intensity until a plateau was reached. The transition to a plateau indicates saturation of fluorescent DNA binding to the particle. The data were fitted with

$$F = \frac{q}{2Kc_p}\left((c_iK + c_pNK + 1) = \sqrt{(c_i + c_pNK + 1)^2 - 4K^2c_ic_pN}\right) \tag{1}$$

which follows from equillibrium binding. Here $c_i$ is the imager concentration, $c_f$ the particle concentraiton, $K$ is the sequence depedent hybridization constant and $q$ and $N$ are fitting parameters corresponding to the fluophore efficiency and the number of DNA strands per particle, respectively.

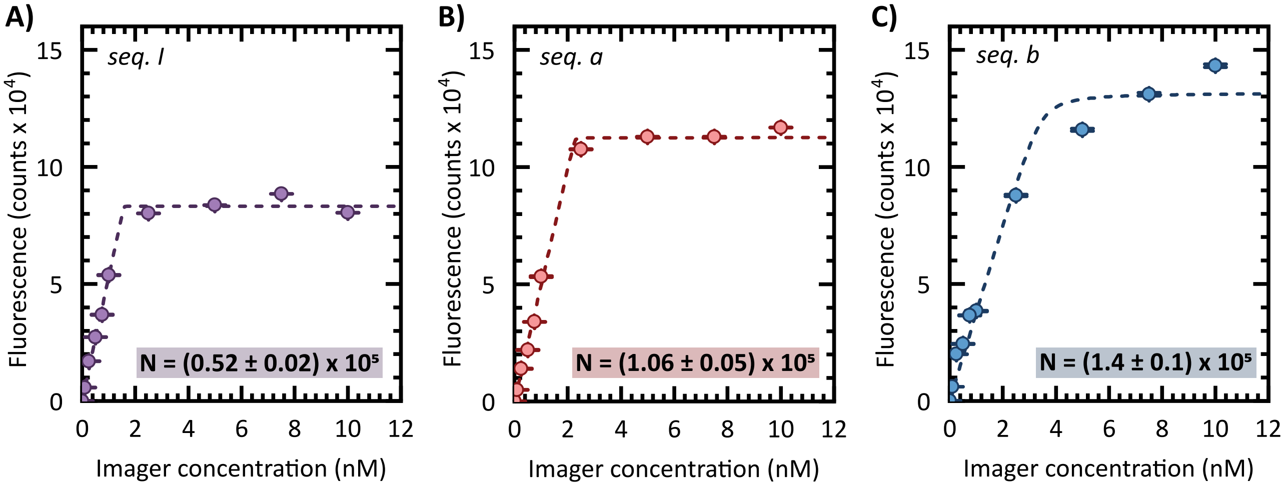


Fig. 1 The plots show the increase in fluorescence intensity as a function of the concentration of fluorescent (imager) DNA added to the suspension for (A) sequence $I$, (B) sequence $a$ and (C) sequence $b$. The data were fitted using Equation 1, and the number of DNA strands per particle, $N$, was determined from the onset of the plateau.

## 4 Flow cytometry data (fluorescence histograms) of multispecific particles prepared by DBCO click chemistry

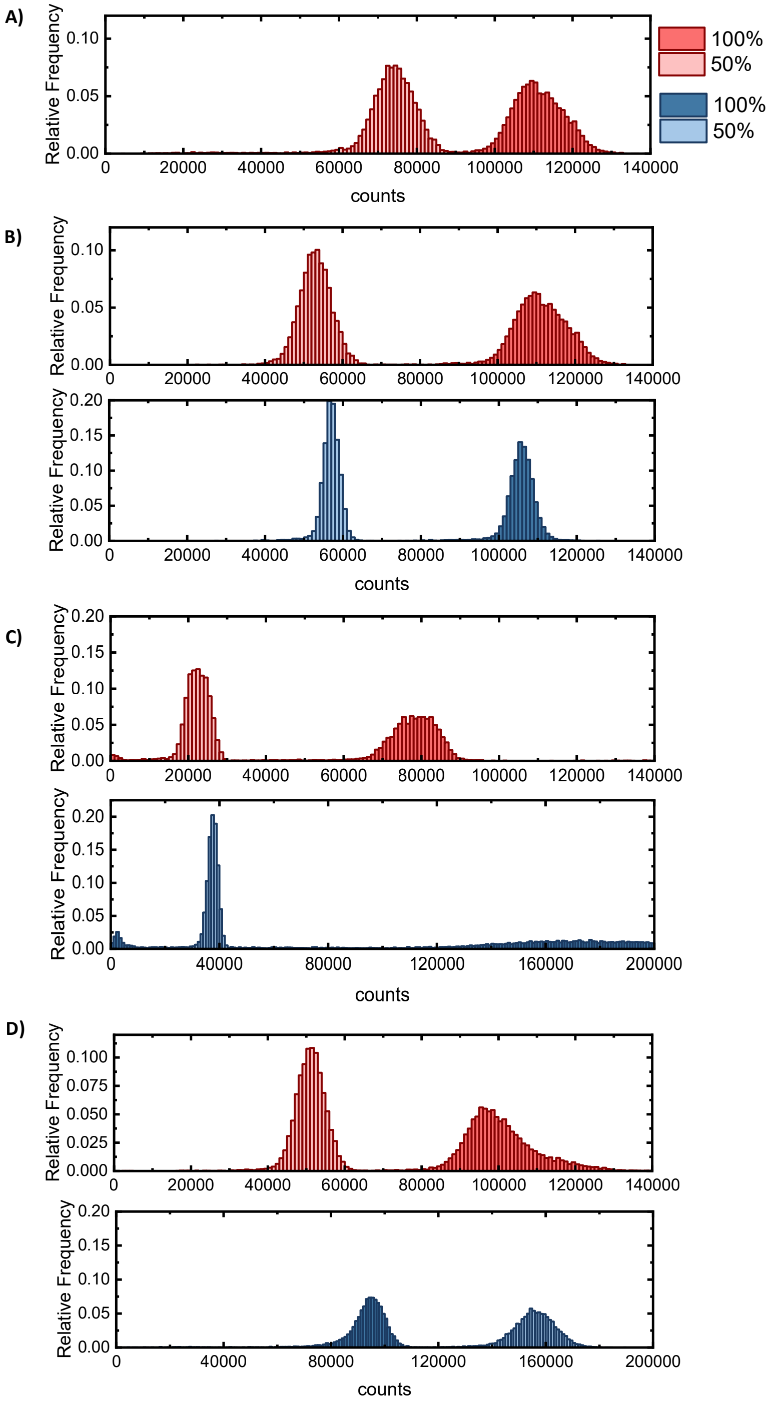


Fig. 2 Representative fluorescence histograms for four sequence combinations targeting a grafting fraction of 0.5, shown alongside reference particles with a grafting fraction of 1. Fluorescense histograms are given for both seq *a* (red) and the second sequence that is varied (blue); (A) seq. a + poly-T (not measured), (B) seq. a + seq. b (short), (C) seq. a + seq. b (long), (D) seq. a + seq. c (short, no poly-T spacer). Histograms reflect fluorescence from 10,000 individual particles measured after hybridization with fluorescent complementary strands. Differences in histogram width and intensity arise from variations in fluorescent labels and hybridization energies; notable cases include the broad reference distribution in (C) and enhanced fluorescence in (D), likely due to local sequence environment. Because the grafting of the two sequences is coupled, interpretation focuses on the red-channel data, which used a constant imager and exhibited Gaussian-like distributions, indicating that deviations from target grafting fractions are not attributable to particle-to-particle heterogeneity.

## 5 Fluorescence measurements of primer-exchange-reactions

### 5.1 Flow cytometry histograms during a primer-exchange reactions

To quantify the rate of new DNA domain growth on particle-grafted DNA (Fig. 3C), we took aliquots at different time points during a primer-exchange reaction. The particles were then hybridized with an excess of complementary fluorescently labeled DNA, and the increase in fluorescence was measured over time for 10,000 particles. An example for a primer-exchange reaction using sequence *a* at a template concentration of 10 nM (Fig. 2C, 4) is shown in Fig. 3.

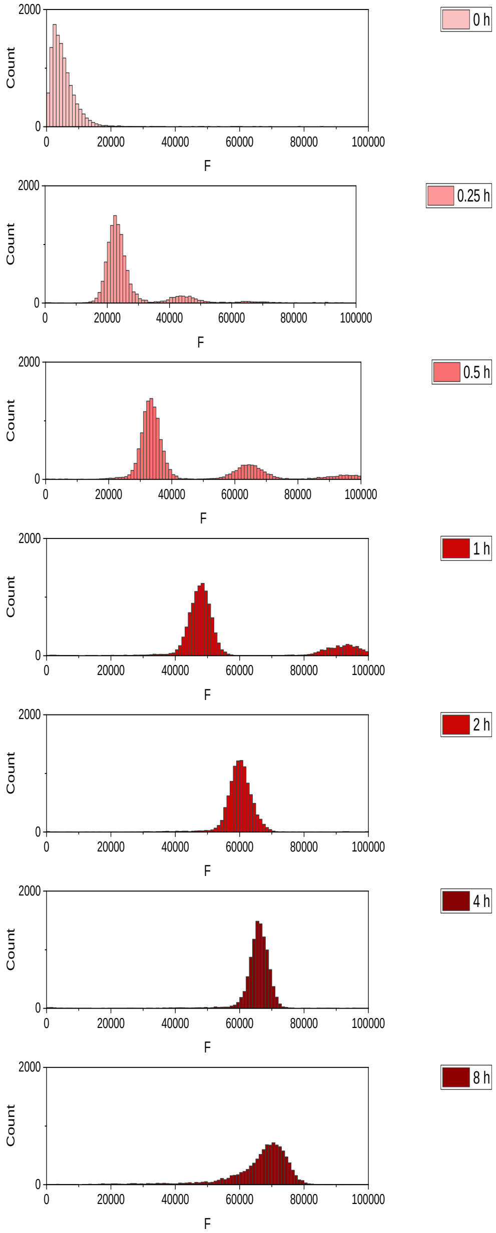


Fig. 3 Histograms show the distribution of fluorescence measured for 10,000 particles hybridized with complementary fluorescent DNA during flow cytometry at different reaction times. Typically, two Gaussian peaks are observed: a main peak and a smaller, higher-fluorescence peak that can be attributed to larger particles formed by aggregation during sample preparation. From the main peak, we extract the average fluorescence for each condition and monitor the increase over time.

### 5.2 Fluorescence increase over reaction time for primer-exchange reaction for different template concentrations

Fig. 4 shows the averaged fluorescence signal measured over 10,000 particles, plotted as a function of reaction time. These values were obtained from the fluorescence histograms measured by flow cytometry (Fig. 3). Here, the fluorescence reflects the number of fluorescent DNA sequences bound to the particles, which in turn corresponds to the number of DNA groups extended with the new sequence $a$. Regardless of template concentration, the fluorescence increase can be described by

$$F = F_{sat}(1 - exp(-kt))$$

. This equation was fitted to the experimental data to obtain the template-concentration-dependent rate $k$ and the saturation fluorescence $F_{sat}$, which represents the maximum grafting density on a particle. Interestingly, although all reactions were started from the same batch of particles, we observed that $F_{sat}$ varies slightly, indicating some variation in the final grafting densities. The grafting fractions shown in Fig. 2C are defined relative to the maximum grafting for each batch:

$$f_a = F/F_{sat}$$

.

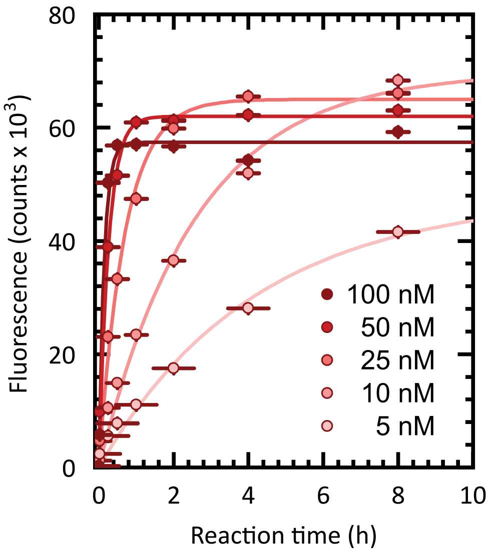


Fig. 4 Fluoresent signal measured over 10000 particles with flow cytometry plotted against the reaction time for several template concentrations and fitted to obtain values for the rate $R$ and saturation fluorescence $F_{sat}$.

## 6 Primer-exchange reaction rates in triplicate

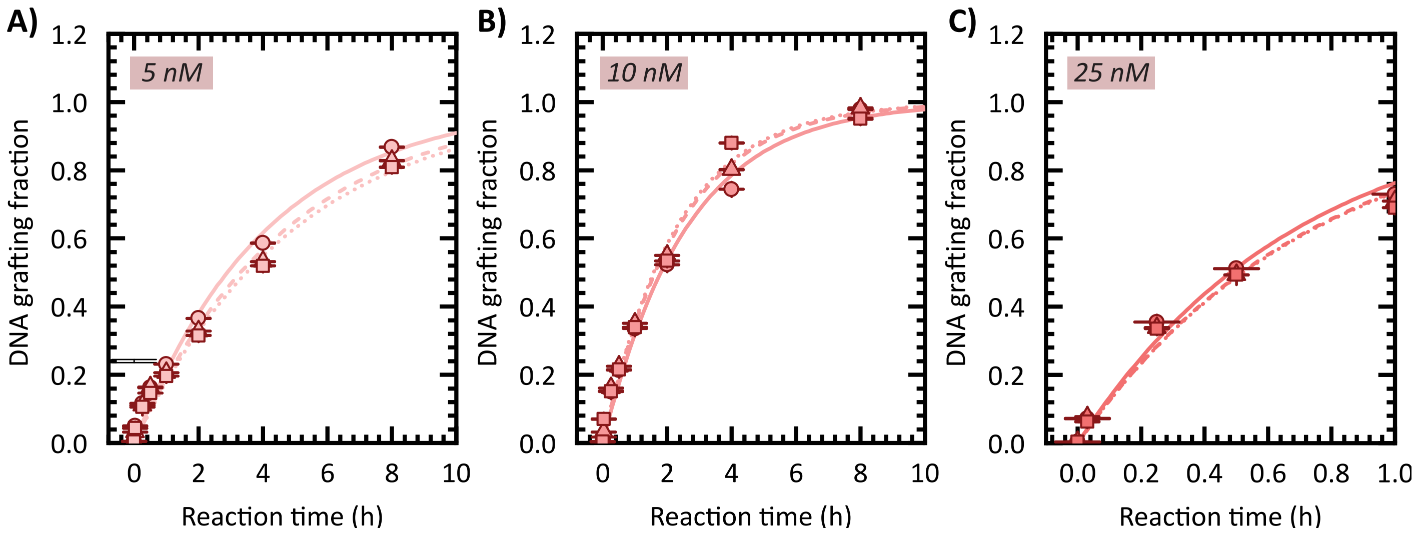


Fig. 5 Reaction rates of the primer-exchange reaction measured in triplicate for template concentrations of (A) 5 nM, (B) 10 nM and (C) 25 nM, all for sequence *a*. Small variations in rate were observed under identical conditions, likely arising from differences in enzyme activity. These variations introduce deviations of a few percent in the achievable grafting fraction and are most relevant when reactions are not run to completion. In multi-template primer-exchange reactions, both templates are expected to be affected equally.

# 7 Primer-exchange reaction kinetics

## 7.1 Template concentration dependent reaction rates for sequences $a$ and $b$

Reaction rates for the primer-exchange reactions were measured for both sequence $a$ and sequence $b$ at template concentrations ranging from 5 to 100 nM. For both sequences, the reaction rate increases approximately linearly with template concentration, consistent with the trend discussed in Fig. 3C. We initially observe faster polymerization rates for sequence $b$ (blue) than for sequence $a$. This difference can be attributed to sequence dependence of the polymerization reaction[1–3] or to differences in template strand release and rebinding kinetics. Interestingly, at higher template concentrations, no further increase in the reaction rate is observed for sequence $b$. We interpret this as the regime in which template release and rebinding are no longer rate-limiting, and the polymerization step itself becomes the rate-limiting process. The fact that this regime appears for sequence $b$, but not for sequence $a$ within the tested concentration range, is consistent with the hypothesis that the faster rates of sequence $b$ arise from more rapid template turnover. In that case, saturation of the turnover process would occur at lower template concentrations. In the context of preparing multispecific particles via PER, we advise avoiding high template concentrations. In multi-step reactions, high reaction rates amplify deviations from the target composition due to limited time precision. In competitive reactions, the plateau in the rate makes it difficult to fine-tune the relative reaction rates of different sequences.

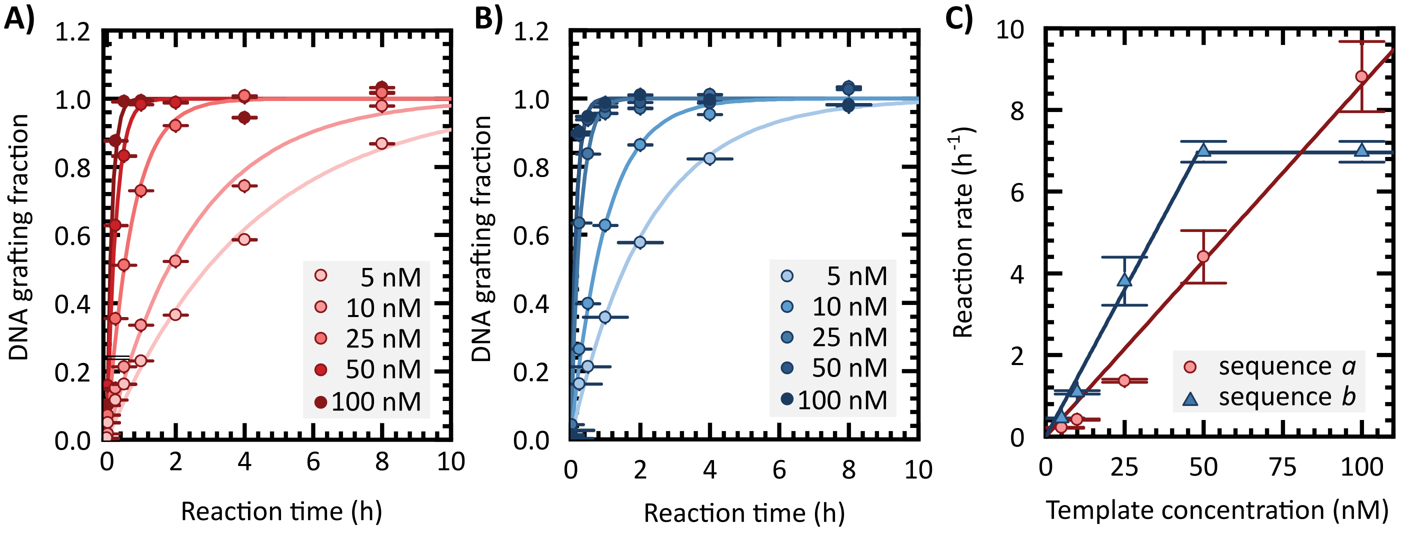


Fig. 6 (A) Reaction rates measured for all template concentrations of sequence $a$. (B) Reaction rates measured for all template concentrations of sequence $b$. (C) Reaction rates calculated from the fits of panels A and C. Reaction rates increase linearly with template concentration, as shown by the blue data at some point the reaction rate flattens to a maximum, we hypothesize that this is because at high concentration the reaction is no longer limited by the template concentration but by the time required to grow new DNA domains. To control the composition of multispecific coatings it's essential to stay away from these high template concentrations.

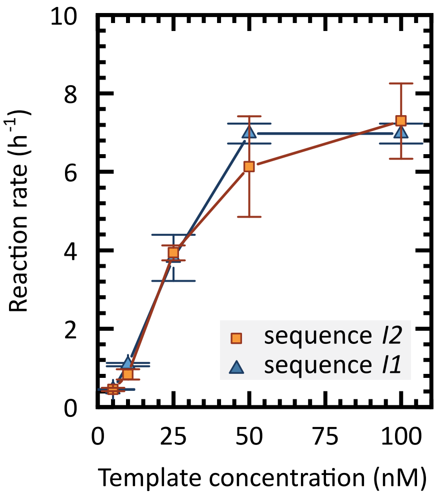


Fig. 7 Rates were measured for two different starting domains (I1: ACTTCACTT and I2: CTCCTAATTC), where in both cases sequence bbb was grown. The measured rates show that the growth rate is independent of the exact starting domain, at least when the initial sequences have similar length and binding energy.

### 7.2 Collapse on master curve upon rescaling

Because the reaction rate $k$ scales linearly with template concentration (Fig. 6C), the conversion curves obtained at different template concentrations (Fig. 6) can be collapsed onto a single master curve by rescaling time with the measured rate constant $kt$. Specifically, plotting the grafting fraction as a function of the dimensionless time causes the data from all template concentrations to overlap (Fig. 8). This collapse indicates that the primer-exchange reaction follows the same underlying kinetic mechanism across the explored concentration range. The template concentration primarily sets the timescale of the reaction, without altering the functional form of the growth dynamics.

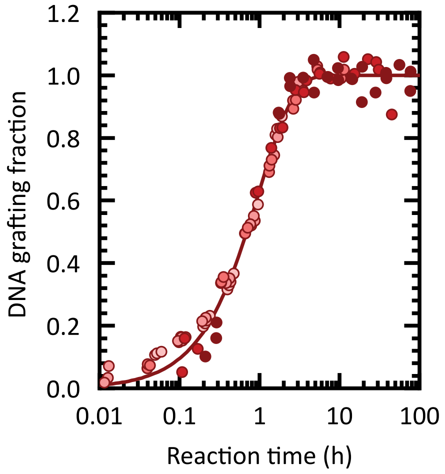


Fig. 8 Rescaled primer-exchange reaction kinetics. Grafting fraction as a function of rescaled time $kt$ for different template concentrations. The rate constant $k$ was obtained from exponential fits to the fluorescence increase. Data collapse onto a single master curve upon rescaling.

## 8 Multi-step and multi-template reactions

Fig. 9 shows the increase of grafting fraction of sequences $a$ and $b$ during a multi-step PER and competitive PER reaction. Both approaches were designed to target grafting fractions of $f_a = f_b = 0.5$. In the multi-step reaction (Fig. 9A), sequence $a$ slightly exceeded the target, reaching $f_a = 0.52$. After initiation of the second step, sequence $b$ reached a final grafting fraction of $f_b = 0.31$, below the intended value. In the simultaneous multi-template reaction (Fig. 9B), both sequences increased at comparable rates but again reached unequal final grafting fractions, with $f_a = 0.42$ and $f_b = 0.30$. The deviation from the target values is likely related to a lower overall maximum grafting density of the particle batches used for the multispecific coatings compared to the reference particles used to calibrate the reaction conditions.

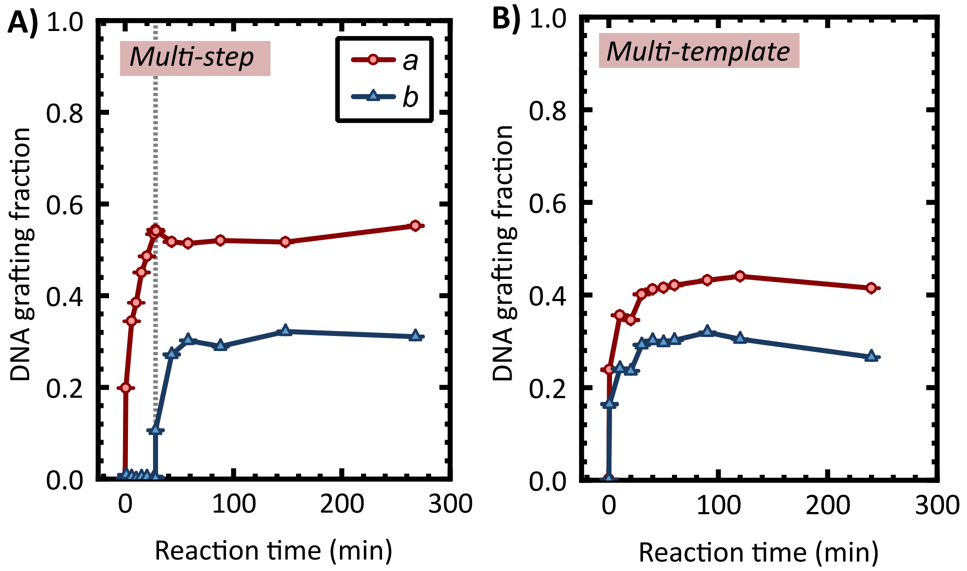


Fig. 9 (A) Grafting fractions of sequences $a$ and $b$ during a multi-step PER reaction. Sequence $a$ is grown first; after 28 min (dashed line), the reaction is stopped and a second reaction is initiated to grow sequence $b$ on the remaining sites. (B) Grafting fractions of sequences $a$ and $b$ during a multi-template PER reaction in which both sequences are grown simultaneously.

## 9 Binding energy calculations

To calculate the free energy of hybridization of strands in free in solution using NUPACK[4] – using T = 20 C, a salt concentration of 1M NaCl and DNA concentration of 1 $\mu$M. For the sequence combination *Ia* and $a'$ this gave a binding free energy of –13.98 kcal/mol, and for the sequence combination *Ib* and $b'$ this gave a binding free energy of –12.94 kcal/mol. Assuming that DNA strands bind independently, in the absence of significant steric or cooperative effects, the total interaction free energy can be approximated as $\Delta G_{total} = n_{bonds} \Delta G_{hyb}$. Under this approximation, we calculate that, to equalize the total binding energies, the grafting fractions must satisfy $f_a = \frac{13.98}{12.94+13.98};= 0.52$ and $f_b = \frac{12.94}{12.94+13.98};= 0.48$. ]

## 10 Structural analysis for microscopy images in Fig. 5

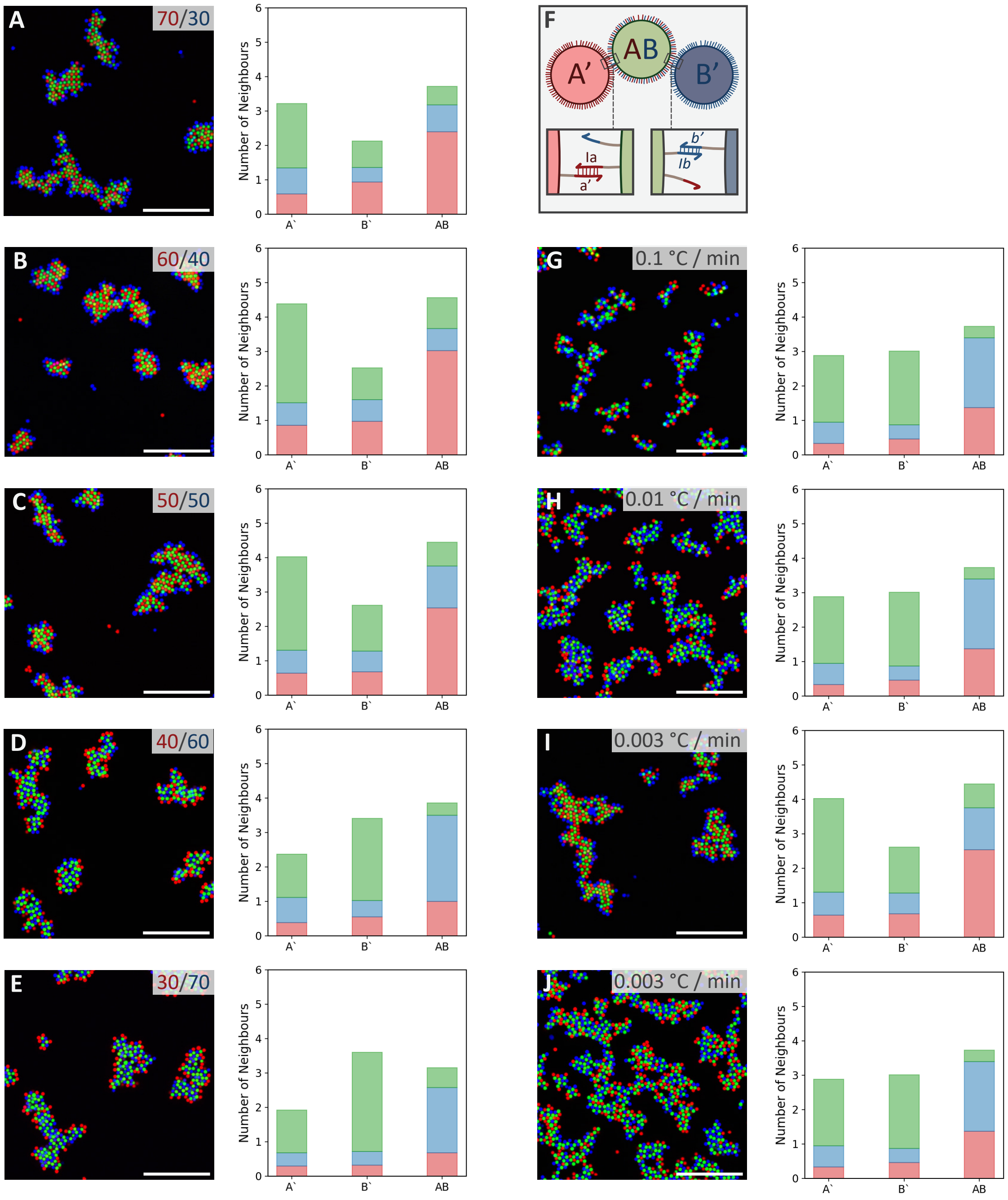


Fig. 10 Various self-assembled structures of multispecific particles (green) with their complementary binding partners (red and blue), together with the mean number of neighbours of each particle type. (A–E, I) Structures formed for different grafting fractions of the two sequences on the multispecific particle during a cooling ramp of 0.003 °C/min. The mean number of neighbours of multispecific particles ($AB$) shows a slight preference for binding to either $A'$ or $B'$, accompanied by a lower number of neighbours for the less-preferred particle type, indicating core–shell-like structures. (F) Schematic illustration of the interactions between multispecific particles ($AB$) and their complementary binding partners ($A'$ and $B'$), highlighting the possible binding configurations. (G–J) Structures formed from 50/50 multispecific particles and their complements under different cooling ramps. The resulting structures depend on the cooling rate, with the order of assembly varying between conditions; in some cases, identical samples under the same cooling ramp yield different structures due to unavoidable sample-to-sample and temperature variations. In panels (G, H, J), the mean number of neighbours of multispecific particles ($AB$) shows equal binding to both $A'$ and $B'$, accompanied by similar neighbour counts for both species, indicating mixed structures. Scale bars represent 20 $\mu$m.

## 11 Additional microscopy images for figure 5

### 11.1 Control experiments of multispecific particles with both binding partners

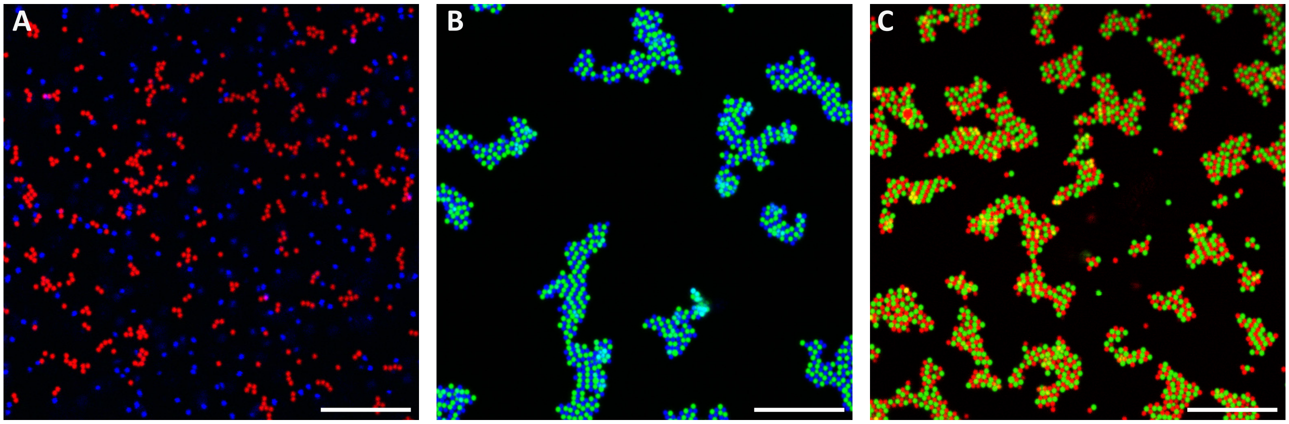


Fig. 11 (A) Complementary particles coated with sequences a' (red) and b' (blue) mixed in the absence of the multispecific binding partner do not interact. Occasional small clusters of red particles are observed and attributed to weak, non-specific DNA hybridization. (B,C) Multispecific particles (blue) coated with grafting fractions of $f_a = 0.53$ and $f_b = 0.47$ for both sequences a and b were mixed separately with binding partners coated with the complementary sequences a' (red) and b' (blue), respectively. Both samples were then cooled slowly, in steps of 0.3 °C per hour, to induce crystallization. The microscopy images show the resulting structures after this temperature ramp, revealing crystalline order. These results indicate that multispecific particles retain their ability to crystallize with either binding partner. Scalebars equal 20 $\mu$m.

## 11.2 Self-assembly structures at different temperatures during annealing

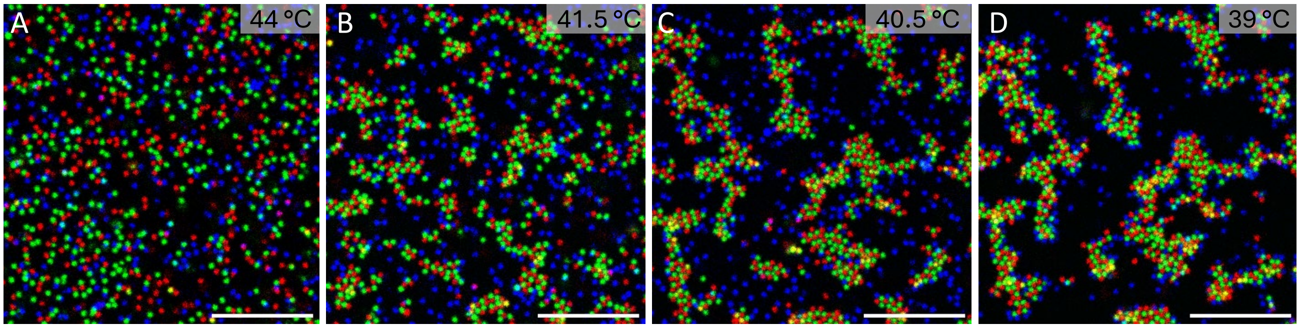


Fig. 12 Various self-assembled structures of multispecific particles (green) when mixed with two complementary particle types (red and blue). The particles were functionalized with equal amounts of both DNA sequences, and the sample corresponds to the data presented in Fig. 5. Snapshots were acquired during a temperature ramp from 44 °C to 39 °C at a cooling rate of 0.1 °C/min. At high temperatures, all particles are present as freely diffusing singlets. Upon decreasing the temperature, clusters begin to form stepwise: initially, assemblies composed of green and red particles emerge, followed approximately 1 °C later by the incorporation of blue particles into the preformed clusters. Upon reheating, the system reversibly returns to the singlet state. This reversible clustering, together with sequence-dependent melting temperatures, demonstrates that particle binding is DNA-mediated and that assembly temperatures differ between sequences. Scalebars equal 20 $\mu$m.

### 11.3 Self-assembly structures of additional grafting densities

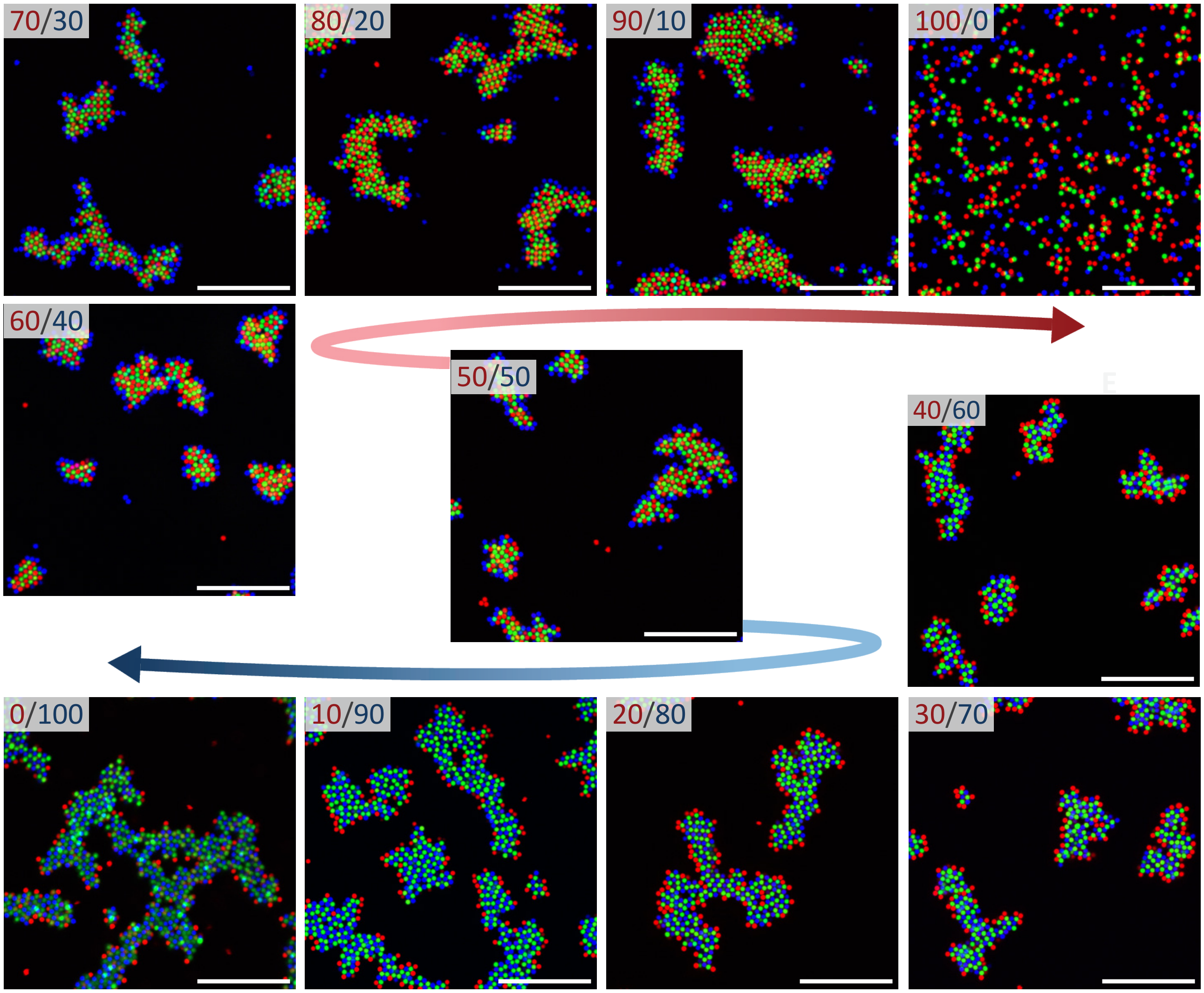


Fig. 13 Various self-assembled structures formed by multispecific particles (green) mixed with their complementary binding partners (red, blue). The structures arise from samples cooled at 0.003 °C/min and prepared with different grafting fractions of the two sequences on the multispecific particles. Core–shell architectures are observed, with the order of assembly switching depending on the grafting fractions. At higher grafting densities, crystalline domains appear within the inner cluster.Scalebars equal 20 $\mu$m.

## 11.4 Self-assembly structures under faster cooling rates

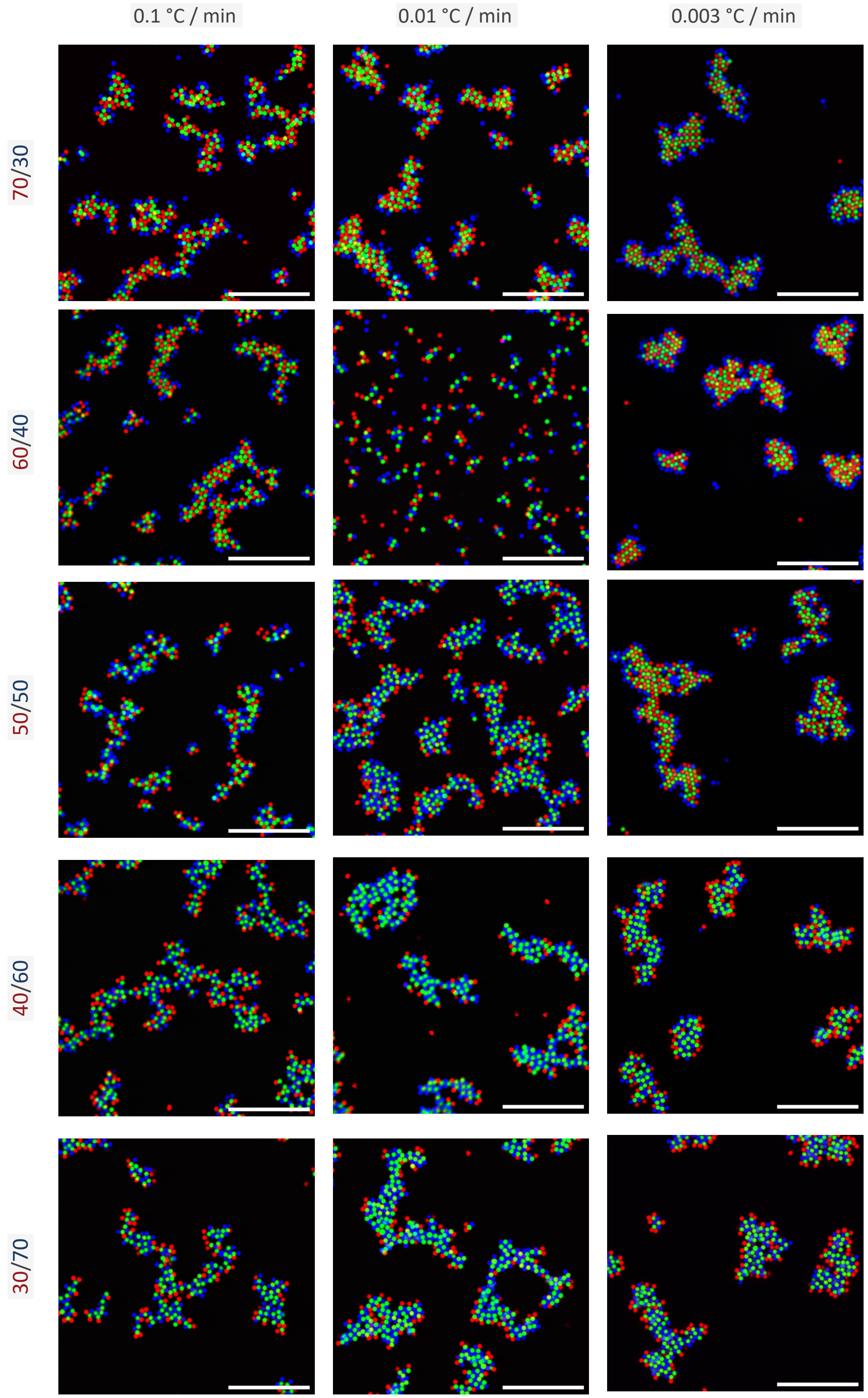


Fig. 14 Structures formed from multispecific particles (green) and their complementary partners (red, blue) under different cooling ramps and for various DNA grafting ratios. Faster cooling rates produce more disordered, mixed structures, with the switch in assembly order occurring gradually. Slower cooling ramps yield more ordered, compositionally separated clusters, with a sharper transition in assembly order as the grafting ratio is varied. Scalebars equal 20 $\mu$m.

## Notes and references


1 Kenneth A. Johnson. Conformational coupling in dna polymerase fidelity. *Annual Review of Biochemistry*, 62(1):685–713, June 1993.

2 Jocelyn Y. Kishi, Thomas E. Schaus, Nikhil Gopalkrishnan, Feng Xuan, and Peng Yin. Programmable autonomous synthesis of single-stranded DNA. *Nature Chemistry*, 10(2):155–164, February 2018.

3 Pierre Murat, Guillaume Guilbaud, and Julian E. Sale. DNA polymerase stalling at structured DNA constrains the expansion of short tandem repeats. *Genome Biology*, 21(1):209, December 2020.

4 Joseph N. Zadeh, Conrad D. Steenberg, Justin S. Bois, Brian R. Wolfe, Marshall B. Pierce, Asif R. Khan, Robert M. Dirks, and Niles A. Pierce. NUPACK: Analysis and design of nucleic acid systems. *Journal of Computational Chemistry*, 32(1):170–173, January 2011.
